# Auditory Processing in High-Functioning Adolescents with Autism Spectrum Disorder

**DOI:** 10.1371/journal.pone.0044084

**Published:** 2012-09-12

**Authors:** Anne-Marie R. DePape, Geoffrey B. C. Hall, Barbara Tillmann, Laurel J. Trainor

**Affiliations:** 1 Psychology, Neuroscience & Behaviour, McMaster University, Hamilton, Ontario, Canada; 2 Psychiatry and Behavioural Neurosciences, McMaster University, Hamilton, Ontario, Canada; 3 Rotman Research Institute, Baycrest Hospital, Toronto, Ontario, Canada; 4 Lyon Neuroscience Research Center, Auditory Cognition and Psychoacoustics Team, CNRS-UMR 5292, INSERM U1028, Université Lyon 1, Lyon, Rhône-Alpes, France; Goldsmiths, University of London, United Kingdom

## Abstract

Autism Spectrum Disorder (ASD) is a pervasive developmental disorder including abnormalities in perceptual processing. We measure perception in a battery of tests across speech (filtering, phoneme categorization, multisensory integration) and music (pitch memory, meter categorization, harmonic priming). We found that compared to controls, the ASD group showed poorer filtering, less audio-visual integration, less specialization for native phonemic and metrical categories, and a higher instance of absolute pitch. No group differences were found in harmonic priming. Our results are discussed in a developmental framework where culture-specific knowledge acquired early compared to late in development is most impaired, perhaps because of early-accelerated brain growth in ASD. These results suggest that early auditory remediation is needed for good communication and social functioning.

## Introduction

Autism Spectrum Disorder (ASD) is a pervasive developmental disorder that includes abnormalities in perceptual processing [1], language and communication [2], and social interaction [3]. Although a diagnosis on the basis of social behavior and language delay is often not possible until a child is at least 3 years old, recent evidence suggests that perceptual processing differences are apparent in the infancy period [4]–[7]. Indeed the early perceptual capacities of those with ASD may set up a cascade of developments that contribute to the poor social skills and perseveration seen at older ages. ASD is associated with a particular processing style in which local stimuli details are very well processed, sometimes at the expense of global processing [8]. For example, those with ASD tend to perform better than those without ASD on tasks such as finding visual embedded figures [9]–[11]. Auditory processing is of particular interest in this regard as there are reports of both hypersensitivity to sound [12]–[17] and hyposensitivity to sound [15], [18]–[21], both of which could interfere with the quality of communicative exchanges and thereby interrupt language and communication development. In this paper, we measure several aspects of auditory processing in speech and music with the purpose of developing an auditory profile that characterizes high-functioning ASD.

From a developmental standpoint, we might expect that aspects of speech and music learning that typically occur early in development, such as perceptual reorganization for native phonemic categories and musical metrical structure, might be particularly affected in ASD. There is evidence that brain growth is accelerated in ASD early in development (particularly 6 to 24 months of age) and slows sooner compared to normal development [22]–[24]. There are also reports of both an overdevelopment of short-distance neural connectivity [25]–[27] and reduced long-distance neural connectivity [27]–[33]. Such irregular patterns of connectivity would be expected to contribute, among other things, to abnormal auditory perceptual processing [34]. Although the precise implications of these neurodevelopmental abnormalities for perception are not known, they might lead to less categorical perception of speech and musical sounds, more attention to less relevant sound features, a focus on local compared to global features, and less specialization for the particular language or musical system in one’s native environment.

With respect to speech processing, research shows that those with ASD activate the middle and inferior temporal gyri bilaterally when listening to speech sounds, whereas controls show more left hemisphere activation [35]. Furthermore, listening to speech sounds produces activation outside of speech-specific areas, such as the brainstem, cerebellum, cingulum and posterior parietal that is not seen in controls [35]. Thus, those with ASD produce abnormal brain activation patterns that involve recruiting suboptimal neural networks for speech sounds. Other research shows that those with ASD show a reversal of the typical left-right brain size asymmetry for areas important for speech and language processing, including the left inferior frontal gyrus (or Broca’s area) and the posterior left superior temporal gyrus (or Wernicke’s area) [36]. Taken together, those with ASD appear to respond differently to speech sounds than controls. It may be that there is a lesser degree of differentiation in people with ASD between the neural pathways that they use to process speech versus environmental sounds, compared with typically developing individuals.

We have created a battery of tests to examine: (1) ability to filter out sounds that are irrelevant to a task and focus on those that are relevant, (2) sensitivity to phonemic categories relevant to the language spoken, (3) multisensory integration of auditory and visual information in speech, (4) propensity to use an absolute pitch code, (5) development of specialization for the metrical categories used in the musical system in the native environment, and (6) internalization of the rules of tonal harmony used in the musical system in the native environment. Here we measure each of these abilities in high-functioning adolescents with ASD in comparison to controls and examine whether there are correlations between these abilities that could reflect general auditory processing styles in ASD. The rationale for including each of these specific tests is outlined in the following paragraphs.

### Test 1

The ability to filter out sounds that are irrelevant to a task and focus on those that are relevant is critical for being able to follow a conversation in a noisy environment, as most environments contain several objects emitting sounds that overlap in time. Questionnaire-based research on this topic suggests that those with ASD score high on items that tap into auditory filtering problems [37]–[41]. For example, in the Short Sensory Profile, parents tend to rate statements “doesn’t respond when name is called but you know the child’s hearing is okay” and “distracted or has trouble functioning if there is a lot of noise around” as describing their child with ASD [42]. Behavioral tasks [43], [44], auditory cortical event-related potentials (ERPs), and auditory brainstem responses (ABRs), the latter two derived from electroencephalogram (EEG) recordings [45], [46]–[48], indicate filtering problems in ASD. Those with ASD require a higher signal-to-noise ratio than controls in order to perceive speech in pink noise, noise from a competing talker or noise with the long-term spectral shape of speech [43], [44]. Furthermore, those with ASD show less evidence of segregating incoming sounds into the auditory objects that compose them [46], and greater difficulty ignoring distracting sounds in peripheral spatial locations [45] compared to normal controls. In the present paper, we measure the ability to ignore one speech stream while attending to another, a task that adults need to perform virtually every day. Specifically, we measure the signal-to-noise ratio needed to perceive sentences presented to one ear while ignoring simultaneous sentences presented to the other ear.

### Test 2

Efficient processing of speech relies on perceiving speech sounds according to the phonemic categories of the language spoken. Typically developing infants are able to discriminate between all possible speech contrasts at 6 months of age, but by 12 months, infants have already become specialized for categorical contrasts used in their native language and have difficulty discriminating contrasts used in foreign languages but not their native language [49]–[51]. Synaptic pruning appears to underlie perceptual specialization [52]. Given the evidence of abnormal neural connectivity in development in ASD, there is reason to suspect that phoneme perception may develop to be less language-specific in ASD than it is in controls. Interestingly, in typically developing infants, a context involving human social interaction is much more effective than an equal amount of exposure in a non-interactive context for phonemic learning to occur [53]. Thus, early social deficits in ASD might also be hypothesized to lead to poorer specialization for the native language. People with ASD also find faces less salient than do people without ASD [54]–[56], so infants who go on to develop ASD might have impoverished visual input during the process of learning native phoneme categories. The only experimental evidence of phoneme categorization in ASD comes from a study that examined foreign speech contrasts [57]. Although this study found that there were no group differences in performance, it cannot address the issue of specialization because it did not compare perception of foreign and native speech sound categories. Here we measure specialization by comparing perception of sounds from a foreign language that map onto one versus two sound categories in the native language.

### Test 3

Typical listeners integrate information about speech from different sensory modalities. In particular, visual information from the eyes and mouth is combined with auditory information to produce a single percept of the sounds produced by a speaker. Experimental evidence of multisensory integration in speech comes from studies on the McGurk effect, in which participants are asked to report what they hear when presented with audio and visual inputs that are incongruent. For example, when presented with a visual “ga” and an audio “ba”, people report hearing a third percept “da” which represents a fusion between what they see and what they hear [58]. A few studies show that those with ASD tend to be less susceptible to this illusion than those who are typically developing, as evidenced by fewer fused responses [59]–[61]. In an experimental task where the auditory and visual information was not in conflict and so no third percept was produced, Smith and Bennetto [62] examined multisensory integration by comparing speech perception performance with the audio alone and with the audio and visual information together. The results showed that both groups performed better in the bimodal than unimodal condition, but the ASD group benefited less than the control group from the addition of visual information [62]. Additional research suggests that those with ASD are less accurate lip-readers than controls [60]–[62], although one study did not find any deficits in this area [59]. Critically, some of these studies indicate that these deficits might contribute to problems with the audio-visual integration of speech [60], [61]. Here we compare those with ASD and controls on their integration of audio and visual information in speech when the information from these two modalities is in conflict. We additionally examine the relative contribution of lip-reading to this integration, or lack thereof, in ASD by including a visual only (lip-reading) condition.

### Test 4

Pitch information is crucial for processing both prosody in speech and melody in music. In humans, pitch is processed in two basic ways. One way is to use a relative code where the pitch distances between tones are encoded, such that a melody is recognized regardless of the starting note or pitch register. By at least as young as 6 months of age, infants process pitch using this type of code [63]–[66]. The second way is to use an absolute code in which individual tones are recognized without relying on an external reference. The ability to name absolute pitches in isolation is extremely rare in adults, being found in less than 5 out of every 10,000 individuals [67], [68]. Although sometimes considered a gift, absolute pitch may hinder melodic and prosodic perception because it focuses attention on single tones instead of on the entire melody, word or phrase. A number of studies indicate that absolute pitch processing may be more prevalent in ASD [69]–[72]. However, these studies primarily tested participants who were explicitly familiar with music reading and Western musical nomenclature, as the tasks required naming notes in Western notation. Here we measure the prevalence of absolute pitch processing in ASD using a task that does not require explicit knowledge of musical structure and can therefore be used in non-musicians with and without ASD.

### Test 5

Just as exposure to a language early in development leads to perceptual processing specialized for that language, exposure to a musical system, such as Western tonality, results in specialized perceptual processing for that musical system [73], [74]. Such specialization occurs for both the rhythm (meter) structure [75]–[77] and the pitch (tonal) structure [64], [78], and this musical enculturation is essential for appreciation of the music in one’s culture. Meter involves the perceptual extraction of an underlying pulse that can be broken down into different hierarchical beat patterns [75]. Simple meters involve strong and weak beat durations that form simple ratios (e.g., 2∶1), whereas complex meters involve strong and weak beat durations that form complex ratios (e.g., 3∶2) [73], [79]. Typically developing 6-month-old infants are able to detect changes in simple meters that are common in Western music as well as changes in complex meters that are rare in Western music but common in Eastern European music [75]. Similar to the enculturation that occurs in language, infants exposed to Western music lose the ability to perceive complex meters in favour of simple meters that are common in their environment by 12 months of age [75], [80]. For the same reasons that we suspect less specialization for native phonemic categories in individuals with ASD, namely early developmental differences in brain development and social interaction, we expect that metrical perception would be less Western-specific in those with ASD compared to controls. To our knowledge, no experimental data to date has addressed how those with ASD perceive metrical categories. Here we measure specialization by comparing perception of native and foreign metrical structures that were implemented in short musical sequences.

### Test 6

As with meter, experience with Western music during development leads to perceptual specialization for the rules of tonal harmony in that musical system. Between 4 and 7 years of age, typically developing children have acquired some implicit knowledge of Western harmonic structure [74], [78], [81], [82]. Chord sequences follow preference rules such that a sequence sets up expectations in enculturated listeners for which chords are likely to come next, and these expectations can be measured implicitly with reaction times [83]–[86]. In a typical implicit paradigm, chord sequences are presented, half of which end with expected chords and half with unexpected chords. Response times on an indirect task, which does not require judging the sequence itself, but rather just making a speeded judgment on the final chord (the target), such as a timbre identification task, are compared for sequences with expected and unexpected endings. For example, when the final chord of the sequence functions as the tonally related (supposed to be expected) tonic chord, response times on this chord were faster than when the final chord functions as the less-related (supposed to be less-expected) subdominant chord [82], [83], [85], [86]. The construction of these experimental stimuli allows the conclusion that listeners have acquired knowledge about the regularities of the Western tonal system, which provides the basis to develop expectations for the final chord type (favoring the tonic over the subdominant). This cognitive priming interpretation contrasts with a sensory priming interpretation. Sensory priming would predict faster processing for the subdominant chord based on the advantages of repetition priming (the repeated presentation of the subdominant inside the sequence) which does not require tonal knowledge. One study asked participants with ASD to report aloud if chord sequences sounded complete or not [87]. Those with ASD were found to process chord sequences similarly to controls in this respect [87]. This study measured accuracy, but reaction times might be a more sensitive measure to reveal group differences. Here we examined harmonic priming by measuring both accuracy and reaction time, and by using an indirect task (i.e., participants were not asked to explicitly judge the sequences’ endings, but were instead asked to quickly discriminate between two target timbres).

In sum, we have developed a battery of tests that measures auditory perceptual processing across the domains of speech (filtering, phoneme categorization and multisensory integration) and music (absolute pitch, meter categorization and harmonic priming). We expect that relative to those who are typically developing, those with ASD would focus more on surface details and less on relative or categorical aspects that are often most important. Our goal is to produce an auditory profile that characterizes high-functioning ASD that could help to inform remediation programs related to auditory processing in communication and social functioning.

## Methods

### Participants

This research was approved by the McMaster University Research Ethics Board and conforms to the Canadian Tri-Council Policy Statement on Ethical Conduct for Research Involving Humans. Written informed consent was obtained from all participants as well as their parents. A total of 54 adolescent male participants (*M* age = 14.8 years, *range* = 11 to 18 years) were tested, 27 with ASD (15 Asperger’s syndrome and 12 High-Functioning Autism) and 27 showing typical development (controls). Among the ASD group, 16 participants had diagnoses (ADOS and ADI) [88], [89] that were completed at the Offord Centre in Hamilton and 11 confirmed through a letter from their family doctor (diagnosis outside of Hamilton) because ADOS and ADI scores were not available. None of the participants in the control group had a family member with ASD, but 11 out of 27 participants in the ASD group (41%) reported a family member with this disorder. All participants were monolingual English speakers, who had similar chronological age and years of musical experience (see Table 1). Among participants who had some musical experience (19 controls and 16 ASD), their experience was similar across the groups. The control group collectively represented 8 instruments (drums, guitar, piano, saxophone, trumpet, trombone, violin, and recorder), while the ASD group collectively represented 11 instruments (French horn, recorder, trumpet, cello, piano, guitar, harmonica, keyboard, glockenspiel, viola and drums). Participants in both groups were more likely to report learning how to play these instruments in the context of a music class at school or from a family member at home instead of through private lessons. Interestingly, the reported estimate of absolute pitch was higher in the ASD group (22%) than the control group (4%), despite the fact that none of the participants had been tested previously for this ability. With respect to family background, both groups reported on average having one sibling, although there was a range of 0 to 4 siblings per household. Finally, most participants identified themselves as being right-handed (74% controls and 85% ASD) rather than left-handed.

**Table 1:** Demographic and background information by group.

	Control	ASD
	Mean (SD)	Mean (SD)
Age, in years	14.6 (2.0)	15.0 (1.7)
Music, in years	2.7 (2.9)	2.3 (2.8)
Forward Digits	10.4 (2.3)	9.6 (2.1)
Backward Digits	5.7 (1.9)	5.6 (2.2)
PPVT, standard	112.3 (9.7)	107.2 (16.4)
Leiter, standard	106.4 (15.4)	99.4 (17.0)

*Note.* Music = Years of Musical Experience; PPVT = Peabody Picture Vocabulary Test; Leiter = Leiter International Performance Scale.

### Procedure and Measures

All participants in the control group were tested at McMaster University. Participants with ASD were either tested at McMaster (*n = *16) or in a quiet room in their own home (*n* = 11) if they lived outside Hamilton. All efforts were made to ensure consistency between testing locations, such that testing in the home was free from distractions. Participants were told that they would be asked to play a series of games using paper and pencil or a laptop computer (Acer Notebook) that was connected to a set of headphones (Sennheiser HAD 200). They were asked to perform to the best of their ability and were assured that they would receive practice trials before starting each game to ensure that they understood the instructions.

After obtaining informed consent, all participants were tested in the same order on the following tasks: Pitch Discrimination (based on [90]), Absolute Pitch (based on [63], [91]), Harmonic Priming (based on [85]), Digit Span Subtest of the Wechsler Memory Scale-III [92], Peabody Picture Vocabulary Test-III [93], McGurk Auditory-Visual Integration Task (based on [58]), Phoneme Categorization (based on [49]), Metrical Categorization (based on [75]), Hearing Thresholds, Competing Sentences Test (based on [94]), and Leiter International Performance Scale [95]. We did not measure full scale intelligence as we were interested in particular skills, such as digit span (as some of our tasks had memory demands) and receptive vocabulary (given the linguistic components involved in our tasks). While participants completed these measures, caregivers were asked to complete a Background Information Form. Those with ASD took approximately 4 hours (2×2-hour sessions) to complete the auditory battery, whereas those in the control group took approximately 3 hours. Participants were compensated $10 for each hour of their time, and received a debriefing statement at the end of the session.

### Background and Baseline Measures

#### Background information form

This parental report contained 16 questions across four areas: demographic information, language exposure, family background and musical training.

#### Leiter international performance scale [95]

This standardized test measures non-verbal intelligence through the use of visualization and reasoning. The four subscales took approximately 30 minutes to complete.

#### Peabody picture vocabulary test-III [93]

This standardized test measures receptive vocabulary through the use of pictures and took approximately 25 minutes to complete.

#### Digit span subtest of the wechsler memory scale-III [92]

The forward digit span portion of this standardized test measures short-term memory and involves repeating back sequences of 1 to 9 digits. The backward digit span portion measures working memory and involves repeating back sequences of 1 to 9 digits in the opposite order to that presented. This subtest took approximately 5 minutes to complete.

#### Hearing thresholds

Thresholds were measured in the right and left ears at 500, 1000, 2000, 4000, and 8000 Hz. Each tone was first presented at 30 dB SPL and adjusted for intensity using a programmable attenuator [96]. Participants were instructed to raise their hand whenever they heard the tone. Following standard audiological assessment procedures, the signal was increased or decreased in amplitude by 2 dB from the previous trial depending on whether the participant was able to detect the tone on the previous trials. The stopping rule for each frequency was three consecutive missed trials. Threshold was measured as the intensity at which a tone for a particular frequency was detected 50 percent of the time. Normal hearing involves an absolute threshold between 0 dB and 20 dB (specifically, 9.5 dB at 500 Hz, 5.3 dB at 1000 Hz, 4.3 dB at 2000 Hz, 8.0 dB at 4000 Hz, and 18.7 dB at 8000 Hz) [97]. This test took approximately 10 minutes to complete.

#### Pitch discrimination [90]

On each of 40 trials, two pure tones were presented separated by 1 sec using Presentation 11.0 [98]. The first tone was always 524 Hz while the second tone was higher or lower in pitch by .25 (8 Hz), .50 (15 Hz), 1.00 (30 Hz) or 2.00 (61 Hz) semitones. Participants were instructed to press the “up” arrow on the keyboard (“A” key) if the second tone was higher in pitch than the first tone and press the “down” arrow on the keyboard (“L” key) if the second tone was lower. The order of trials was randomized across participants. This task took approximately 10 minutes to complete.

### Speech Perception Measures

#### 1. Competing sentences test (based on [94])

This test measured the signal to noise ratio needed to repeat back a simple sentence (5 to 6 words) spoken by a male speaker in one ear while ignoring a semantically related sentence in the other ear. The distracting sentence (the “noise”) was presented at 50 dB above each individual’s threshold at 1000 Hz. A response was counted as correct if it contained at least two words from the target sentence and no words from the distracting sentence. The test was programmed using Microsoft Visual Basic and presented on the laptop computer, connected to a programmable attenuator [96]. The signal was initially presented to all participants at 30 dB above their hearing threshold for 1000 Hz. A Bayesian adaptive psychometric procedure [99] was used such that the test ended when the standard deviation of the signal threshold estimate reached 1.5 dB or less. Performance was indicated by the signal to noise ratio (SNR) (signal in dB – noise in dB) at which performance was 50 percent correct. This test took approximately 15 minutes to complete.

#### 2. Phoneme categorization (based on [49])

On each trial, participants heard three 300 msec phonemes in an ABB or AAB format and determined whether the first or last phoneme was different from the other two. All of the speech sounds were from a South African language (Zulu) and spoken by the same female native speaker. Two sets of two phoneme categories were used such that one contrast (24 trials) mapped onto distinct phonemic categories in English (specifically, voiced lateral fricative vs. voiceless lateral fricative) and should therefore be easy for English speakers to discriminate, whereas the other contrast (24 trials) mapped onto the same phonemic category in English (specifically, plosive bilabial stop vs. implosive bilabial stop) and should therefore be more difficult for English speakers to discriminate. On each trial, for each of the three phonemes, one of 6 possible tokens for a given phoneme (matched in duration and fundamental frequency) was chosen randomly with the constraint that the same token could not be used twice in the same trial. The measure of interest was the comparison between accuracy on the one- and two-category mapping conditions. Before starting each test condition, participants received 6 practice trials where they were asked to discriminate between contrasts in the one- and the two-category mapping conditions. We used stimuli from both test blocks in the experimental phase to ensure that participants in the practice phase understood the task instructions. Participants were told to respond as accurately as they could. The task was programmed in Presentation 11.0 [98]. The order of stimulus presentation was randomized across participants. This task took approximately 15 minutes to complete.

#### 3. McGurk task (based on [58])

This computerized task measured the audio-visual integration of speech. On each trial, participants heard and/or saw a face making mouth movements for a consonant-vowel pair (“ba”, “ga”, or “da”) that was produced 6 times at a normal speaking rate. There were three types of audiovisual trials: matched auditory “ba” plus visual “ba” (12 trials); matched auditory “ga” plus visual “ga” (12 trials); and mismatched auditory “ba” plus visual “ga” (24 trials). There were four types of single modality control trials presented after the audiovisual trials: auditory only “ba” (6 trials), auditory only “ga” (6 trials), visual only “ba” (6 trials), and visual only “ga” (6 trials). There were five tokens each of “ba” and “ga”. On each trial, one token was chosen randomly. Participants were asked to indicate what they heard (or saw in the case of visual only trials) by pressing “1” if they heard “ba”, “2” if they heard “ga”, and “3” if they heard “da”. If participants were integrating the audio-visual information in the mismatched trials then they should report hearing “da” (McGurk illusion). The measures of interest were the susceptibility to the McGurk illusion and the relative contribution of lip-reading to multisensory integration. Participants were instructed not to stop looking at the face in the video until she stopped talking, and their behavior was monitored in this regard throughout the task. The task was programmed in Presentation 11.0 [98]. The order of stimulus presentation was randomized across participants. This task took approximately 20 minutes to complete.

### Music Perception Measures

#### 1. Absolute pitch (based on [63], [91])

In the initial control condition, on each trial participants heard a 500 msec piano tone, followed by 16 seconds of silence, followed by a second tone that was either at the same pitch (6 trials) or one semitone higher (3 trials) or lower (3 trials), and indicated whether the two pitches were the same or different. The experimental condition was identical except that the silent period contained a 500 msec pause, then 15 interfering tones (randomly chosen on each trial but ranged within an octave such as A2/110 Hz to F3/175 Hz), followed by a pause of 8 seconds and then the final tone. The same random order of trials was used for each participant. The stimuli were presented in Windows Media Player 10.0. A perfect score on the experimental condition indicated absolute pitch processing. This task took approximately 15 minutes to complete.

#### 2. Metrical perception

Using the stimuli of [75], on each trial participants were first familiarized for 15 seconds with a melody based on traditional Eastern European folk music that either (4 trials) had a Western-typical simple meter (8 note measure subdivided into 2+2+2+2 250 msec beats) or (4 trials) had a Western-atypical complex meter (7 note measure subdivided into 3+2+2 250 msec beats). Following each familiarization, a 30 second test melody was presented that contained an extra note that either preserved the metrical structure of 8 or 7 beats, or added one beat to it, transforming the simple meter into a complex 9-beat meter or the complex meter into a simple 8-beat meter. Before starting the test condition, participants received 5 practice trials using a familiar melody (“Mary Had a Little Lamb”) to ensure that they understood the instructions. Participants were asked to indicate if the two melodies had the same beat or not. Using a response box, participants pressed “1” if the two melodies were “very well” matched, “2” if the two melodies were “somewhat well” matched, “3” if the two melodies were “somewhat poorly” matched, and “4” if the two melodies were “very poorly” matched. The task was programmed in Presentation 11.0 [98]. The order of stimulus presentation was randomized across participants. This task took approximately 25 minutes to complete.

#### 3. Harmonic priming (from [85])

**Figure 1:**
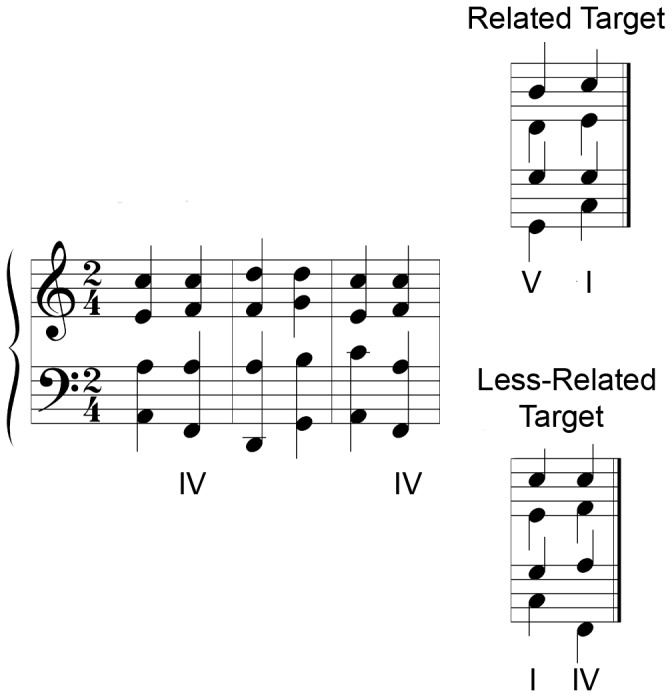
Example stimuli for Harmonic Priming Task (from [**85**]). The 6 chords of the prime context are shown on the left. These are followed by either a dominant (V) to tonic (I, expected) progression or a tonic (I) to subdominant (IV, less expected) progression. Note that the target chord repeats in the prime context for the less-expected subdominant target chord (IV chord), but not for the expected tonic target chord (I chord), ruling out sensory priming explanations of observed processing differences (adapted from [130], Figure 2).

The task was programmed in Presentation 11.0 [98]. In this implicit task participants heard an eight-chord sequence (with the first seven chords, each sounding for 620 ms, defining the prime context, played in piano) on each trial, and indicated whether the 8th chord (the target, duration of 2000 msec) was played in “piano” or “harp” timbre. Unlike in the original experiment by [85], we changed the way that participants made their response by using the terms “piano” and “harp” instead of Timbre A and Timbre B. Importantly, in half the chord sequences, the target chord functioned as the tonic chord (preceded by the dominant chord; 12 trials), whereas in half, it functioned as the subdominant chord (preceded by the tonic chord; 12 trials). There are seven chords that define a key in Western tonal music. Each of these chords is based on a different degree of the scale that forms a hierarchy of stability, depending on the currently installed key. The most stable chord is the tonic (I), and all other chords are perceived in relation to this chord. The next most stable chord is the dominant (V), followed by the subdominant (IV) chord, etc. If participants process these chords according to the regularities of Western tonal music, faster response times are predicted for the tonic targets, which are supposed to be the appropriate, expected ending, than for the subdominant targets, which are also part of the tonality, but are less expected (Figure 1). If participants do not show this cognitive priming effect, they might be influenced by sensory (repetition) priming, leading to faster processing of the subdominant targets, which also occurred in the prime context, than of the tonic targets, which did not occur in the prime context. Note that both predictions require processing the sequences globally as the last two chords are kept constant between the two conditions across the sequence set. Participants initially received 12 practice trials involving single chords to ensure that they understood the instructions of the speeded timbre discrimination task. They were instructed to respond as fast and as accurately as possible and both speed and accuracy were analyzed. The order of stimulus presentation was randomized. This task took approximately 15 minutes to complete.

## Results

### Background and Baseline Measures

The groups were matched in chronological age, (*p* = .43, *M dif* = −.407, 95% CI [−1.435,.620]), in non-verbal intelligence, (*p* = .12, *M dif* = 6.963, 95% CI [−1.884, 15.809]), in receptive vocabulary, (*p* = .18, *M dif* = 5.074, 95% CI [−2.344, 12.492]), and in musical experience, (*p* = .60, *M dif* = .407, 95% CI [−1.128, 1.943]) (Table 1).

#### Digit span

An ANOVA revealed a main effect of condition, *F*(1, 52) = 243.04, *p*<.001, ç^2^ = .82 (*M dif* = 4.296, 95% CI [3.743, 4.849]), with better performance for forward than backward digit span, but no main effect of group (*p = *.39, *M dif* = .444, 95% CI [−.591, 1.480]), or interaction involving group (*p* = .23) (Table 1). In sum, there were no significant differences between control and ASD groups in short-term or working memory for digits.

#### Hearing thresholds

An ANOVA conducted on hearing thresholds revealed a main effect of condition, *F*(1, 52) = 12.24, *p* = .001, ç^2^ = .20 (*M dif* = .98, 95% CI [.416, 1.54]), with better performance in the right than the left ear, and a main effect of frequency, *F*(4, 52) = 14.90, *p*<.001, ç^2^ = .23 with better performance for lower than higher frequencies tested. However, there was no main effect of group and no interactions (all *ps* >.63). In sum, there were no significant differences in hearing thresholds between control and ASD groups.

#### Pitch discrimination

An ANOVA conducted on accuracy scores revealed a main effect of direction, *F*(1, 52) = 6.45, *p* = .01, ç^2^ = .11 (*M dif* = .29, 95% CI [.06,.52]), with better performance for rising than falling pitch, and a main effect of size, *F*(1, 52) = 45.24, *p*<.001, ç^2^ = .47 (*M dif* = .83, 95% CI [.58, 1.07]), with better performance for large than small pitch changes, but no main effect of group or interactions involving group (all *ps* >.05). In sum, control and ASD groups exhibited similar pitch discrimination thresholds.

### Speech Perception Measures

#### 1. Competing sentences task

**Figure 2:**
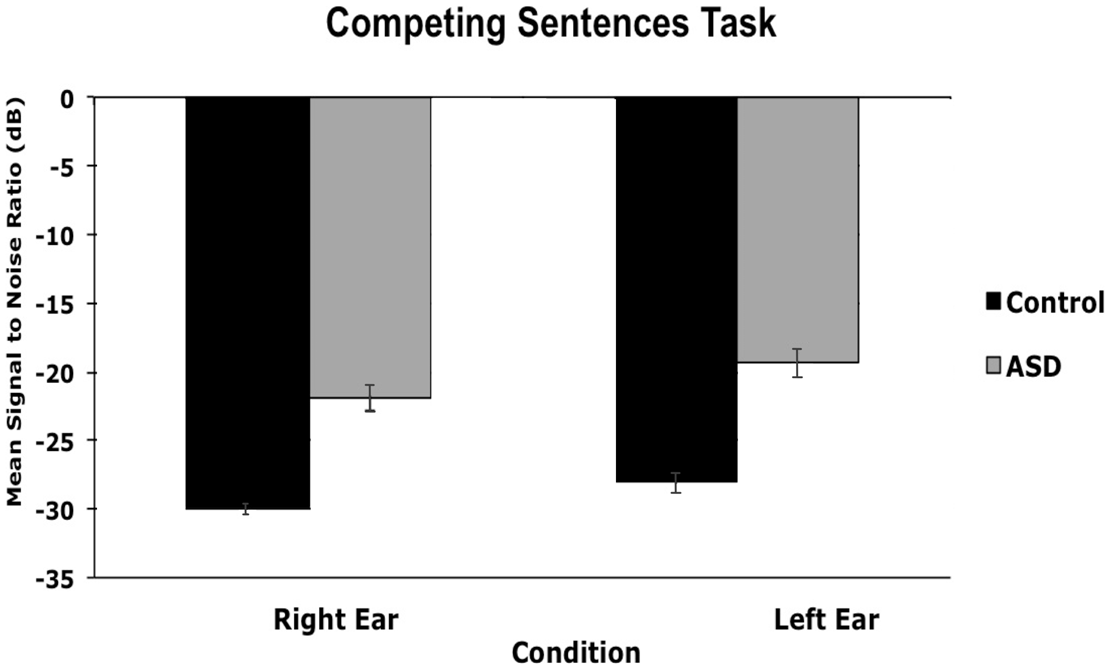
Mean signal to noise ratios for the Competing Sentences Task by group. The signal-to-noise ratio needed to detect the sentence in one ear in the presence of a competing sentence in the other ear are shown on the y-axis. Those with ASD performed significantly worse than controls as evidenced by higher signal to noise ratios.

An ANOVA conducted on mean signal to noise ratio (SNR) (signal in dB – noise in dB) revealed a main effect of ear, *F*(1, 52) = 21.01, *p*<.001, ç^2^ = .31 (*M dif* = 2.23, 95% CI [1.25, 3.21]; Cohen’s *d* = .84), with better performance when the target was presented to the right than the left ear, and a main effect of group, *F*(1, 52) = 52.88, *p* = .000, ç^2^ = .54 (*M dif* = 8.31, 95% CI [6.01, 10.61]; *d* = 1.95), with better performance for the control than ASD group (Figure 2). There was no significant interaction between ear and group (*p = *.47). To determine whether this performance difference for the competing sentences task is explained by receptive vocabulary, we performed an ANCOVA on mean SNR, with receptive vocabulary as our covariate. The ANCOVA revealed a main effect of group, *F*(1, 51) = 47.46, *p* = .000, ç^2^ = .51, but not of ear, *F*(1, 51) = 2.95, *p* = .09, and no main effect of receptive vocabulary (*p = *.37). There was a significant interaction between ear and receptive vocabulary, *F*(1, 51) = 5.44, *p* = .024, ç^2^ = .11, with better performance when the target sentence was delivered to the right than left ear, but no significant interaction between ear and group, *F*(1, 51) = 1.56, *p* = .22. In sum, those with ASD perform worse than controls when required to filter a spatially segregated stream of information, even after accounting for differences in receptive vocabulary.

#### 2. Phoneme categorization

**Figure 3:**
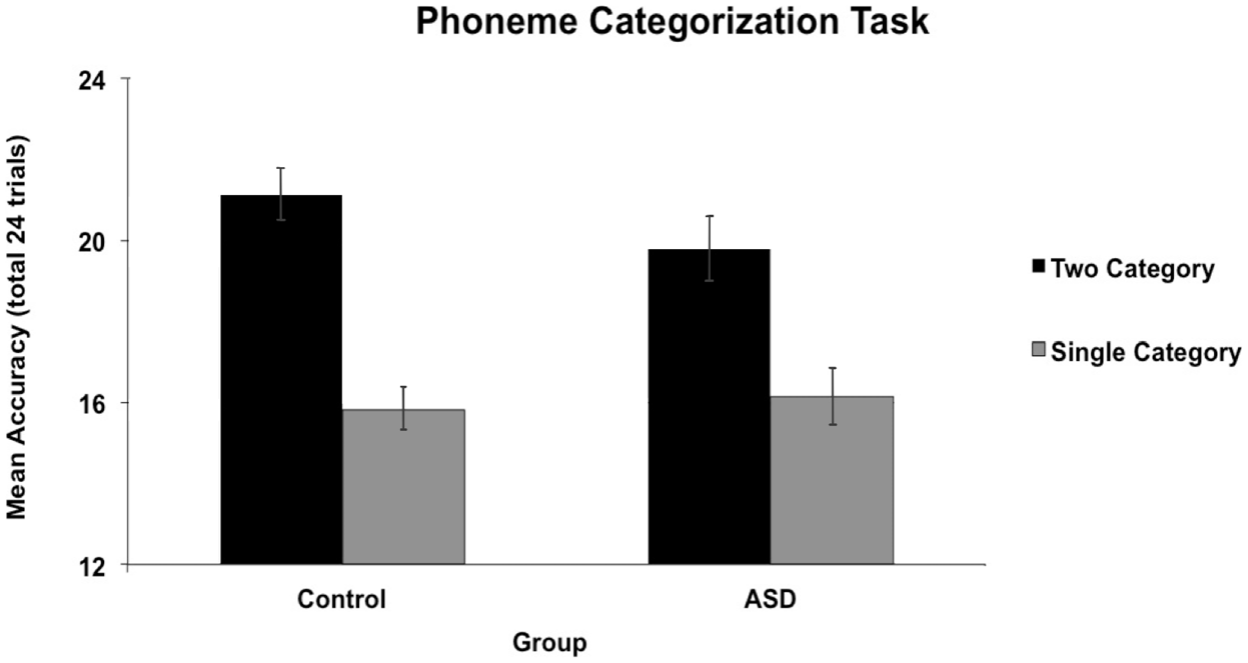
Mean accuracy for the Phoneme Categorization Task by group. In a 3-interval forced choice design, subjects heard three phonemes that fell into two categories in either the pattern ABB or AAB and had to determine whether the middle sound was most similar to the first or last sound. The number correct out of 24 is shown on the y-axis for the cases where the speech sounds fell into one or into two phonemic categories in the native language. Error bars represent standard error of the mean. Group differences were found such that those with ASD showed a significantly smaller difference than controls between the two-category and one-category mapping conditions.

An ANOVA conducted on accuracy scores revealed a main effect of condition, *F*(1, 52) = 138.02, *p*<.001, ç^2^ = .73 (*M dif* = 4.50, 95% CI [3.73, 5.27]; *d = *2.85), with better performance for two-category mapping (native categories) than one-category mapping (foreign categories). The main effect of group was not significant (*p = *.58, (*M dif* = .50, 95% CI [−1.28, 2.28]; *d* = 0.12), but there was an interaction between condition and group, *F*(1, 52) = 4.73, *p* = .03, ç^2^ = .083 (Figure 3) such that those with ASD showed a smaller difference between the two-category and one-category mapping conditions than did controls. Simple main effects using independent samples t-tests revealed no significant difference in performance between groups in the native categories [*t* (52) = 1.56, *p* = .13, *d* = .43] or foreign categories [*t*<1, *d* = .09] conditions. To determine how much of this performance difference for the phoneme categorization task is explained by receptive vocabulary, given that the task involved verbal instructions, we performed an ANCOVA on mean accuracy scores, with receptive vocabulary as our covariate. The ANCOVA revealed no main effect of condition, *F*(1, 51) = 2.70, *p* = .11 (*M dif* = 4.50, 95% CI [3.72, 5.28]), no significance for the main effects of group (*p = *.71, (*M dif* = .34, 95% CI [−1.48, 2.15]) and receptive vocabulary (*p = *.34), and no significant interaction between condition and receptive vocabulary (*p = *.81). However, the important interaction between condition and group remained significant, *F*(1, 51) = 4.63, *p* = .04, ç^2^ = .084. In sum, those with ASD showed less specialization for native speech sound categories than controls, even after accounting for differences in receptive vocabulary.

#### 3. McGurk task (matched trials)

**Figure 4:**
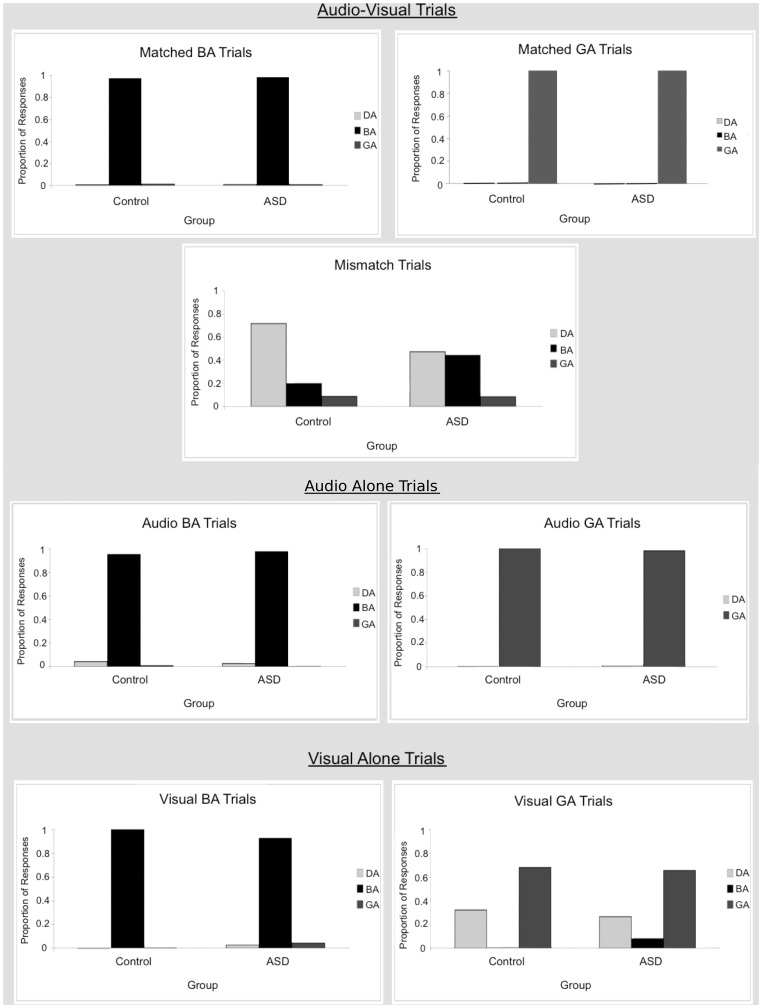
Performance in the McGurk Task by group. Proportion of “ba”, “da” and “ga” responses are shown for each stimulus type. **Audio-Visual Trials.** No group differences were found for the matched audio-visual trials. However, group differences were found for the mismatched audio-visual trials with those with ASD being less likely than controls to report hearing “da” (McGurk illusion) than “ba”. **Audio Alone Trials.** No group differences were found for audio-alone trials. **Visual Alone Trials.** No group differences were found for visual-alone trials.

Performance on matched audiovisual trials was close to or at ceiling for both groups (Figure 4, upper panel). For BA trials, performance was 97.5% correct for the control group and 98.4% correct for the ASD group. Planned independent samples t-tests revealed no significant difference in performance between groups in *ba* responses [*t<*1, *d* = .12], *ga* responses [*t*<1, *d* = .18] or *da* responses [*t*<1, *d* = .10]. For GA trials, performance was 100% correct for both groups. Thus, planned independent samples t-tests could not be performed on these trials because the standard deviation was equal to 0 for both groups. In sum, there were no significant differences between groups in the matched trials.

#### McGurk task (mismatched trials)

Performance was 19.9% correct (i.e., the response was *ba* when presented with the auditory/ba/and visual/ga/) in the control group and 44.5% correct in the ASD group. Planned independent samples t-tests revealed a significant difference in performance between groups for *ba* responses [*t* (52) = 2.89, *p* = .006, *d* = .79] as well as for *da* responses [*t* (52) = 3.01, *p* = .004, *d* = .82], although not for *ga* responses [*t*<1, *d* = .03]. Those with ASD were less likely to integrate audio-visual speech sounds (i.e., less likely to experience the McGurk illusion) as evidenced by fewer *da* responses and more *ba* responses than controls.

#### McGurk task (audio trials)

Performance was high for both groups on auditory alone trials. For BA trials, overall performance was 95.0% correct for the control group and 97.5% correct for the ASD group. Planned independent samples t-tests revealed no significant differences in performance between groups for *ba* responses [*t<*1, *d* = .26], *da* responses [*t<*1, *d* = .20], or *ga* responses [*t* (52) = 1.00, *p* = .32 *d* = .30]. For GA trials, overall performance was 100% correct for the control group and 98.2% correct for the ASD group, precluding performance of t-tests on *ba*, *da* and *ga* responses, but indicating very high performance. In sum, performance was at or near ceiling for both groups and there were no measurable significant differences between groups in performance for the audio only trials.

#### McGurk task (visual trials)

For visual only BA trials, performance was 100% correct for the control group and 92.7% correct for the ASD group. Thus planned independent samples t-test could not be performed on *ba, da and ga* responses, but performance was at or close to ceiling for both groups. For GA trials (more difficult task than BA trials because the place of articulation is at the back of the mouth for/ga/) the groups did not differ in the number of correct (*ga*) responses, 67.8% for the control group and 65.5% for the ASD group [*t*<1, *d* = .08]. Furthermore, the groups did not differ significantly in the number of correct *da* responses [*t*<1, *d* = .20] although there was a significant difference in the number of *ba* responses [*t* (52) = 2.95, *p* = .005, *d* = .80]. Thus, performance was similar across groups in lip reading, although the distribution of errors differed somewhat in the case of GA trials.

In sum, the McGurk Task results indicate that those with ASD were less susceptible to the McGurk illusion, but no group differences were found for auditory alone or visual alone (lip reading) speech sound discrimination.

### Music Perception Measures

#### 1. Absolute pitch test

**Figure 5:**
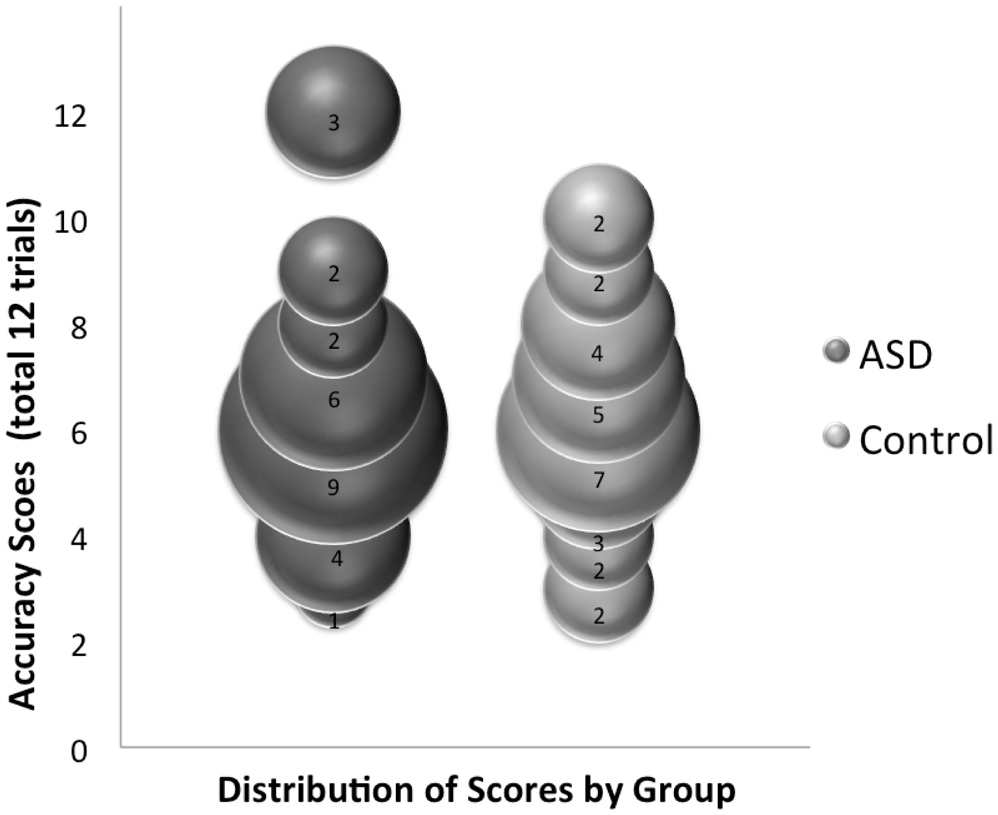
Distribution of absolute pitch scores by group. The size of the bubbles (and the number in each bubble) indicate the number of subjects who obtained each score (number correct out of 12 trials). The column of bubbles on the left represents the ASD data and the column on the right the control data. Three participants with ASD but no controls showed perfect performance, indicative of absolute pitch processing.

**Figure 6:**
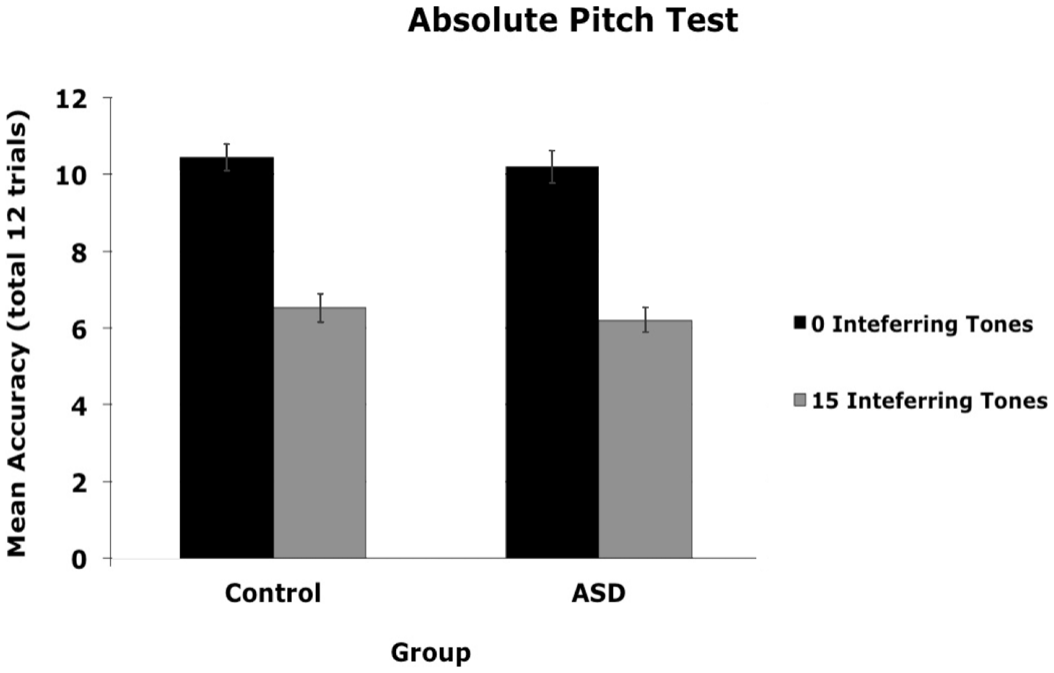
Mean accuracy for the Absolute Pitch Test by group. Mean number correct out of 12 for determining whether two tones had the same or different pitches when there were 0 or 15 interference tones, after removing the three ASD subjects with perfect scores in the 15-tone interference condition indicative of absolute pitch. Error bars represent standard error of the mean. There were no significant differences in performance between groups after removing these participants.

Three participants in the ASD group (3 out of 27 or 11%), but none in the control group (0 out of 27 or 0%), showed perfect performance in this task, indicating absolute pitch processing. To determine whether the ASD sample differed from the normal population, we used the binomial distribution and set the probability of absolute pitch to 5/10,000 [67], [68]. The probability of obtaining 3 or more individuals with absolute pitch from a sample of 27 given *p = *5/10000 is.0000004. We can therefore robustly reject the null hypothesis that our ASD sample was drawn from the normal population (Figure 5). Next, we conducted an ANOVA to determine if there was a significant difference in pitch memory between controls and those with ASD who did not demonstrate absolute pitch (i.e., *n* = 24 ASD, *n* = 27 controls). The results revealed a main effect of condition, *F*(1, 49) = 142.82, *p*<.001, ç^2^ = .75 (*M dif* = 3.96, 95% CI [3.30, 4.63]; *d = *4.84), with worse performance in the presence of interference tones, but no main effect of group (*p = *.50, (*M dif* = .27, 95% CI [−.53, 1.07]; *d* = 0.10), or interaction (*p = *.91) (Figure 6). Together, these results indicate that the prevalence of absolute pitch is higher among those with ASD than in the normal population. However, when those with absolute pitch were removed from the sample, no difference in pitch memory between the ASD and control groups was apparent.

#### 2. Meter perception

**Figure 7:**
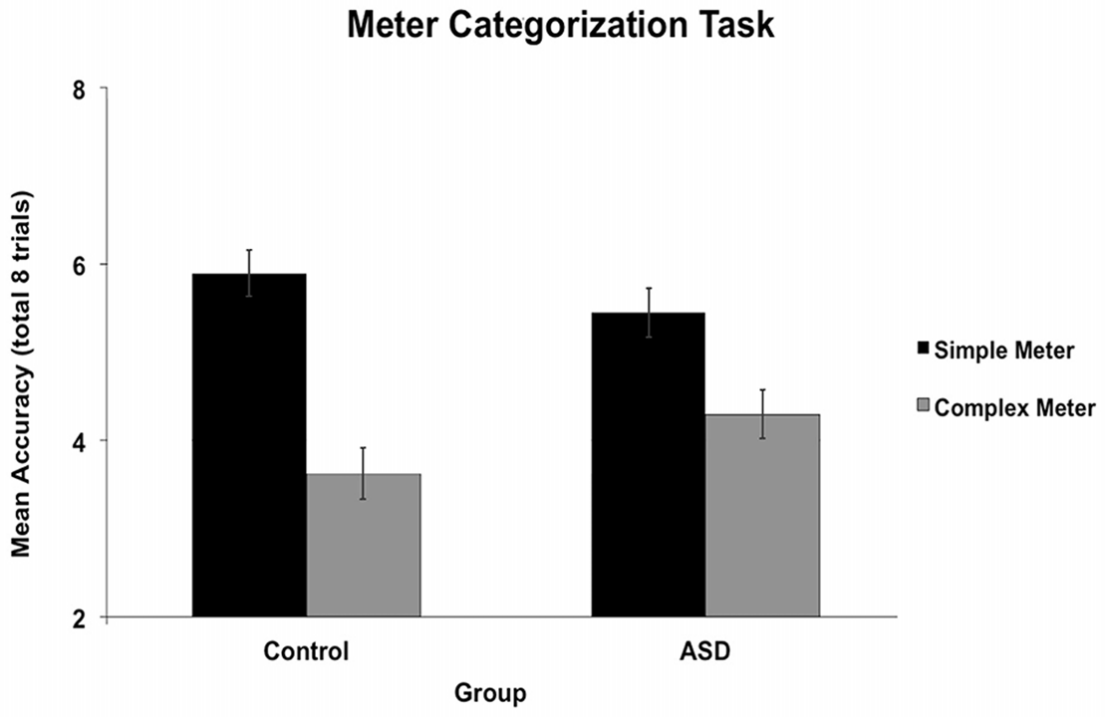
Mean accuracy for the Meter Categorization Task by group. Number correct out of 8 trials is shown on the y-axis. On each trial, it was to be determined whether an excerpt had the same or a different meter compared to a standard excerpt. Error bars represent standard error of the mean. Group differences were found such that those with ASD showed a significantly smaller difference than controls between the simple meter (typical in Western music) and complex meter (rare in Western music) conditions.

An ANOVA conducted on accuracy scores revealed a main effect of condition, *F*(1, 52) = 40.23, *p*<.001, ç^2^ = .44 (*M dif* = 1.70, 95% CI [1.17, 2.24]; *d = *2.61), with better performance for simple (native) than complex meter (foreign) meters, but no main effect of group (*F*<1, *M dif* = .11, 95% CI [−.47,.69]; *d* = 0.07). There was, however, an interaction between condition and group, *F*(1, 52) = 4.28, *p* = .04, ç^2^ = .08 (Figure 7), such that those with ASD showed a smaller performance difference between simple and complex meter conditions than controls. Simple main effects using independent samples t-tests revealed no significant difference in performance between groups in the simple meter [*t* (52) = 1.13, *p* = .27, *d* = .31] or complex meter [*t* (52) = 1.69, *p* = .10, *d* = .46] conditions. In sum, those with ASD showed less specialization for simple meters than controls. To determine how much of this performance difference for the meter categorization task is explained by receptive vocabulary, given that the task involved verbal instructions, we performed an ANCOVA on mean accuracy scores, with receptive vocabulary as our covariate. The ANCOVA revealed no significant effects of condition (*p = *.62, *M dif* = 1.70, 95% CI [1.16, 2.25]), group (*p = *.53, *M dif* = .18, 95% CI [−.40,.77]), or receptive vocabulary, *F*(1, 51) = 1.75, *p* = .19, and the interaction between condition and receptive vocabulary was also not significant. Importantly, the interaction between condition and group remained significant, *F*(1, 51) = 3.86, *p* = .05, ç^2^ = .070. Together, these results indicate that even after accounting for receptive vocabulary, those with ASD still show a smaller difference in performance between simple (native) and complex (foreign) meter compared to controls.

#### 3. Harmonic priming task

**Figure 8:**
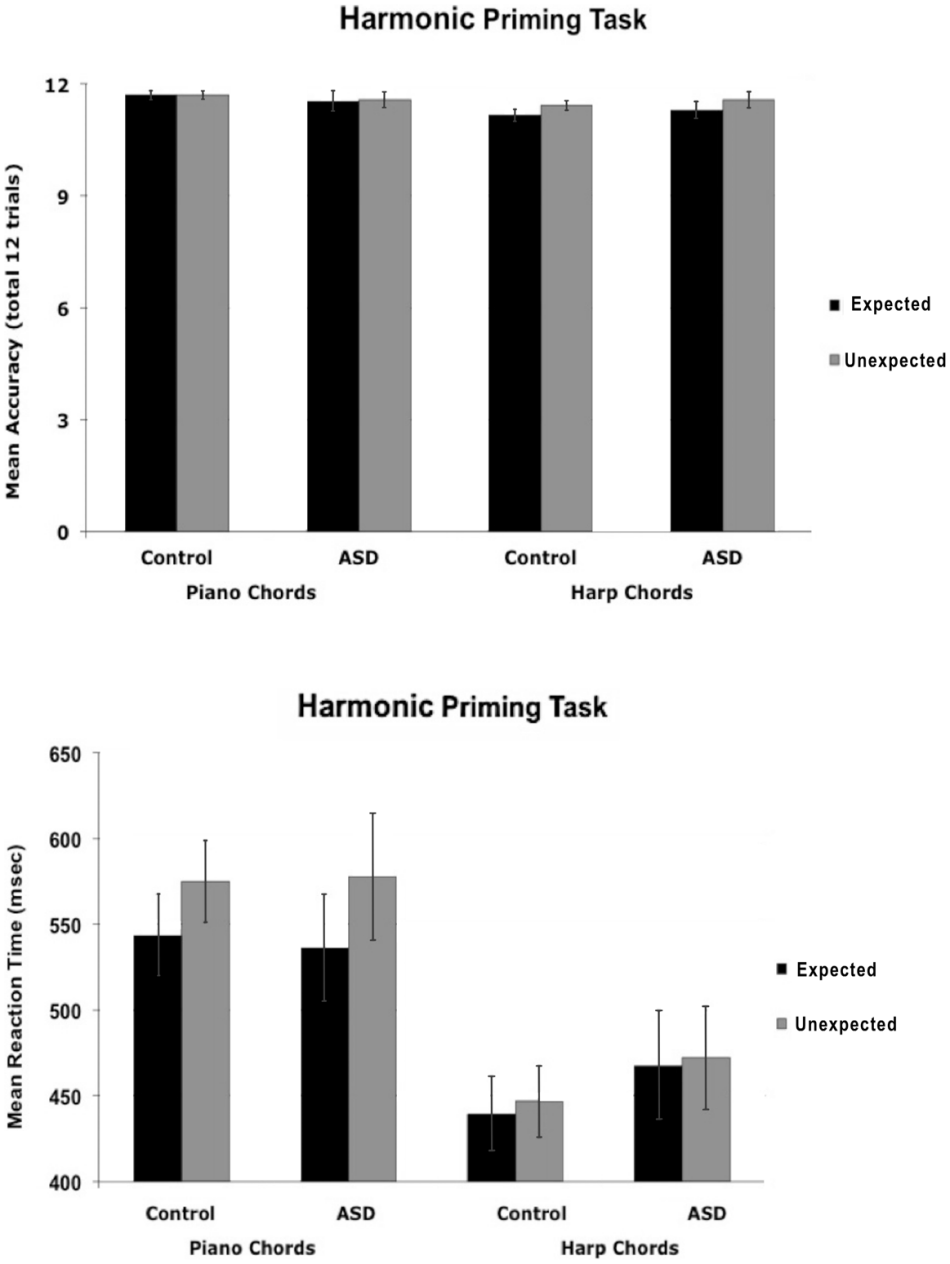
Performance in the Harmonic Priming Task by group. In this implicit task, subjects determined whether the last chord in a sequence was in piano or harp timbre. **A.** Performance on the 12 trials was very high for chords that were expected and for chords that were not expected, with no significant group differences. **B.** No significant group differences were found in reaction time performance. Both groups responded faster to the expected tonic target chords than to the less expected subdominant target chords.

Due to technical problems, the data are missing for 1 participant in the control group and 5 participants in the ASD group. To ensure that the groups were matched on accuracy, we conducted a 2×2×2 ANOVA on accuracy scores with harmonic target type and timbre as within-participant factors and group as a between-participants factor. The only significant effect was the main effect of timbre, *F*(1, 45) = 10.62, *p* = .002, ç^2^ = .19, *M dif* = .26, 95% CI [.10,.42]; *d = *.66, with both groups performing more accurately for piano chords than for harp chords (Figure 8a).

When calculating the response time performance for each participant, only correct responses were included. Response times that were less than 250 msec or greater than 2,500 msec were excluded from the analyses, which are considered to be conservative cutoffs for outliers for reaction time data [100]. These reaction times occurred infrequently (13 out of 2 256 responses) and accounted for less than 1% of total responses. Additionally, one participant with ASD was removed from the final sample because of mean reaction times that were 4 standard deviations slower than the group means.

A 2×2×2 ANOVA conducted on correct response times revealed a main effect of harmonic target type, *F*(1, 45) = 9.68, *p* = .003, ç^2^ = .18, *M dif* = 21.42, 95% CI [7.55, 35.28]; *d = *.36, with faster performance for expected tonic chords than unexpected subdominant chords, and a main effect of timbre, *F*(1, 45) = 79.242, *p*<.001, ç^2^ = .64, *M dif* = 101.45, 95% CI [78.49, 124.40]; *d = *1.69, with faster performance for harp than piano chords, but no main effect of group (*F<*1). There were also no interactions involving group (*p*>.21; Figure 8b). There was an interaction between timbre and harmonic target type, *F*(1, 45) = 3.95, *p* = .05, ç^2^ = .08. Thus two 2×2 ANOVAs were conducted by group on reaction times for piano and harp endings separately. The ANOVA on piano endings revealed a main effect of harmonic target type, *F*(1, 45) = 11.86, *p* = .001, ç^2^ = .21 *M dif* = 36.56, 95% CI [15.18, 57.95]; *d = *.58, with faster performance for expected targets than unexpected target chords, but no main effect of group (*F<*1, *M dif* = 2.23, 95% CI [−77.45, 81.90]; *d = *.02), and no interaction (*F<*1). An ANOVA conducted on reaction times for harp endings revealed no significant main effects or interactions (all *F<*1). Thus, the harmonic priming effect occurred only for piano endings. This pattern of results was also found previously [85] and may reflect the fact that the change to harp timbre is very salient such that processing the timbre change occurs faster than processing of the harmonic information, so no (or less) effect of chord type (tonic/subdominant) is typically seen. It should be noted that the two groups in our sample were similar in showing the priming effect for expected harmonic endings when sequences (composed of piano chords) ended with a piano chord but not when they ended with a harp chord. In sum, the piano chords produced harmonic priming with faster reaction times for the expected than unexpected chord endings, but this performance was the same across control and ASD groups.

### Correlations between Tasks

We examined how receptive vocabulary, non-verbal intelligence, and processing small pitch changes were related to the tasks in the battery using Pearson correlations across our entire sample (*N* = 54). We found that receptive vocabulary was only related to performance on the McGurk task. Specifically, it was related to visual alone BA trials (*r* = .40, *p* = .003) and to *ga responses* on mismatched trials (*r* = −.34, *p* = .012). Non-verbal intelligence was significantly related to simple meter processing (*r* = .29, *p* = .03), processing small pitch changes (*r* = .38, *p* = .005), and lip-reading for the visual alone GA trials (*r* = −.30, *p* = .03). Pitch change processing showed the highest number correlations with other battery tasks. Specifically, being able to detect small pitch changes was related to native speech sound processing (*r* = .29, *p* = .03), simple meter processing (*r* = .29, *p* = .03), absolute pitch processing (*r* = .36, *p* = .009) and working memory for digits (*r* = .37, *p* = .007). Detecting small pitch changes was also related to reaction times for expected (*r* = −.51, *p* = .000) and unexpected (*r* = −.31, *p* = .03) piano chord endings, and to reaction times for expected (*r* = −.41, *p* = .004) and unexpected (*r* = −.55, *p* = .000) harp chord endings. In sum, these correlations show that pitch processing in particular may underlie performance on several of the tasks in the auditory battery.

We also performed Pearson correlations on the same variables using only data from the ASD group (*n* = 27). Here we found a similar pattern of results with the exception that the correlation between receptive vocabulary and responding “ga” on mismatched trials was no longer significant (*r* = −.29, *p* = .14), along with the correlation between detecting small pitch changes and reaction times for unexpected piano chord endings (*r* = −.34, *p* = .14). However, receptive vocabulary was still related to visual alone BA trials (*r* = .42, *p* = .03) for the ASD group. Non-verbal intelligence was also significantly related to simple meter processing (*r* = .39, *p* = .04), processing small pitch changes (*r* = .41, *p* = .04), and lip-reading in the visual alone GA trials (*r* = −.50, *p* = .007). Being able to detect small pitch changes was related to native speech sound processing (*r* = .46, *p* = .02), simple meter processing (*r* = .39, *p* = .05), absolute pitch processing (*r* = .49, *p* = .01) and working memory for digits (*r* = .42, *p* = .03). Finally, detecting small pitch changes was related to reaction times for expected piano chord endings (*r* = −.60, *p* = .004), and for reaction times for expected (*r* = −.45, *p* = .04) and unexpected (*r* = −.59, *p* = .005) harp chord endings. In sum, a similar pattern of correlations was found for the ASD group as was found for the entire sample.

## Discussion

Relative to typically developing adolescents, we found that adolescents with ASD were impaired on some auditory tasks but not on others, forming a profile by which we can further our understanding of auditory processing in this disorder. In general, the two groups were similar in terms of thresholds for sound detection, short-term memory, working memory, receptive vocabulary and non-verbal intelligence. However, compared to controls, the ASD group showed evidence of filtering problems (our competing sentences task showed that filtering problems persist at the level of speech sentence processing), less integration of auditory and visual information in speech, less enculturation to the phonemic categories of their language, and less enculturation to the metrical categories of the musical system in their environment. In general, those with ASD tended to be more impaired on tasks involving speech than on tasks involving musical sounds. Interestingly, with respect to music, although those with ASD showed less metric enculturation than controls, both groups showed similar enculturation to the harmonic pitch structure of Western tonal music. Although it is possible that the group difference on the meter enculturation task reflects difficulties in understanding the explicit task requirements, this is unlikely as the groups did not differ on receptive vocabulary scores, and no significant relation was found between performance on the explicit meter task and receptive vocabulary.

As discussed in the introduction, neural development in ASD appears to be particularly disrupted early in development, with early accelerated brain growth and disrupted patterns of neuronal connectivity [22], [23], [25]–[27], [30]–[32]. Our results are generally consistent with the idea that skills acquired early in development are more disrupted in ASD. Efficient processing of speech relies on perceiving speech sounds according to the phonemic categories of the language spoken. By 12 months of age, normally developing infants, like adults, have become specialized for the language in their environment, and they have difficulty discriminating foreign phonemic categories that map onto a single category in their native language [49]–[51]. Interestingly, ours was the first study to show less specialization for native-language phonemic categories in adolescents with ASD compared to typically developing controls, and this difference between groups persisted even after accounting for individual differences in receptive vocabulary. These results suggest that native language learning may develop more slowly because perception is less constrained among those with ASD than controls. This finding is also consistent with the idea that those with ASD focus on low-level characteristics of sounds whether or not they are relevant to the task. We did not have access to whether individuals with ASD in our study showed early language delay or not, but it would be interesting for future studies to examine whether the development of native phonemic categories is affected by whether or not language delay is present. Interestingly, early social communication may also play a role in the diminution of specialization for native phonemic categories in ASD. In one study, the ability to discriminate foreign speech categories in infancy was maintained when infants interacted with a live person speaking that foreign language, but not when infants were exposed to recordings of that language [53]. Thus, there may be multiple reasons for less phonemic specialization in ASD. In any case, these results suggest that very early remediation may be needed in order to promote development of optimal speech circuits for language in ASD.

Similar to the acquisition of sensitivity to one’s native phonemic categories, specialization for the metrical rhythm structure of the music system in one’s environment is also seen by 12 months of age. We found, in this first study of metrical enculturation in ASD, that those with ASD were less specialized than controls for processing rhythms with simple meters typical of Western music compared to complex meters. This difference could not be explained by amount of musical experience in terms of formal music training as the groups did not differ on this variable. Interestingly, poor socialization early in development may also impair native rhythmic acquisition. Few species can entrain to an auditory beat, and all those who do so appear to be vocal learners [101], [102]. Furthermore, rhythmic entrainment between people during music making has been shown to increase social bonds and promote prosocial behavior [103], [104]. Those with ASD are certainly able to process musical rhythms, but a lack of cultural specialization and social motivation may mean that they experience music somewhat differently from typically developing individuals. At the same time, it should be pointed out that children and adults with ASD appear to perceive emotion in music similarly to normal controls [70], [105].

Everyday experience with Western music during normal development also leads to perceptual specialization for tonal pitch structure [73], [74]. Sensitivity to harmonic structure develops rather late. Some implicit knowledge of harmonic structure can be seen by ages 4 to 7 [74], [78], [81], [82], and explicit judgments emerge between 6 and 12 years of age [106]. Interestingly, we found that those with ASD were similar to controls in showing faster responses to expected tonic chords than to unexpected subdominant chords. Importantly, accuracy was also equivalent in both groups. Thus, the later developing skill related to harmonic structures appears to be relatively spared in ASD, consistent with the idea that the brain is most abnormal early in development. This finding is also generally consistent with the research of Heaton and colleagues [107]–[109], which suggests that musical pitch processing is more spared than speech processing in those with ASD. We extended these previous studies, however, by using an implicit task and examining both accuracy and reaction times.

Consistent with previous literature [69]–[72] we found a high instance of absolute pitch processing in our population with ASD (11% compared to 0% in the control group). Unlike tests for absolute pitch used in previous studies, which required participants to have musical training because the tasks involved naming notes according to Western notational conventions, we used a task that did not require formal musical training. Thus, we show that the previous findings also extend to those without musical training. Although absolute pitch is sometimes considered to be a gift, the more complex, but very common, ability to process relative pitch (comparing the pitch distance between two tones) is more important for both music and speech processing because it enables recognition of melodies and prosodic patterns across high or low pitch registers. It is also interesting that relative pitch typically develops early, with evidence that infants at least as young as 6 months recognize melodies transposed to higher or lower pitch registers [63]–[66]. The prevalence of absolute pitch in ASD, then, is consistent with early abnormalities in brain development. It is also consistent with the general prevalence of savant syndromes in ASD, which is about 10% [110]. In the typical population, the presence of absolute pitch is associated with early experience on a fixed-pitch instrument, leading researchers to speculate that it develops when there is a genetic predisposition combined with a particular environment [111]. In our ASD population, there was no evidence of greater musical experience in those with absolute pitch, suggesting that ASD may involve a genetic propensity for absolute pitch.

Heaton [107] argues that absolute pitch in individuals with ASD is acquired differently than in the rest of the population with absolute pitch, and that anatomical features associated with absolute pitch in the normal population, such as the relative size of the planum temporale, are not present in those with ASD. Recent research suggests that in the general population, those with absolute pitch ability show local hyperconnectivity between the posterior superior and middle temporal gyri [112]. Because those with ASD have been shown to have greater short-range connectivity [25], [26], it would be interesting to determine whether the brains of those with ASD and with absolute pitch also show this feature. Although the number of those with ASD who have absolute pitch in our sample is small, they appear to show similar enculturation to Western tonal harmony as those who do not have absolute pitch, suggesting that absolute pitch and harmonic enculturation are separate abilities in ASD. Given that harmonic enculturation likely relies on relative pitch processing, this suggests that individuals with ASD may use absolute pitch processing in one task (pitch memory) and relative pitch processing in another task (harmonic priming), consistent with previous research [71].

Interestingly, when those with absolute pitch were eliminated from the sample, pitch memory was similar in those with and without ASD as measured by the ability to hold the pitch of one tone in mind and compare it to that of a second tone, whether or not there were interference tones in between. Thus we add to the literature on absolute pitch processing in ASD by showing that although it is more prevalent than in the general population, the majority of those with ASD show similar pitch memory performance as those without ASD. This similar performance across groups on memory stands in contrast to the decrements shown by the ASD group in ignoring one speech stream while attending to a second simultaneous speech stream. This latter difficulty persisted in the ASD group even after accounting for receptive vocabulary, which is consistent with other reports of difficulty filtering non-speech stimuli [46]. The ability to group sounds into different perceptual streams develops early, with evidence for segregation of both simultaneous sounds [113] and sequential sounds [114]–[118] during infancy, although this ability continues to improve until 9 to 11 years of age [119]. Poor filtering in ASD in the context of deciphering speech signals, therefore, may have its origins early in development when brain growth and connectivity are abnormal.

Audiovisual integration is also present during the infancy period, both for speech and non-speech stimuli [120], [121]. Consistent with previous literature [59], [62], and the notion that early developing abilities will be particularly impaired in ASD, we found that those with ASD were less susceptible to the McGurk effect, integrating face and sound information to a much lesser extent than those who were typically developing. On the other hand, we found similar performance across groups on auditory alone and visual alone (lip-reading) conditions. We also found a significant correlation between performance on visual alone BA trials and receptive vocabulary, suggesting a link between lip-reading and general language abilities. Interestingly, higher receptive vocabulary scores were also related to a reduced likelihood of responding “ga” on the mismatched trials. Our results suggest that the audiovisual integration deficit found in previous studies cannot be entirely accounted for by differences in lip-reading ability in the absence of sound. It is possible that the reduction of long-range connectivity in ASD results in inadequate integration of auditory and visual information and perhaps a lack of top-down modulation of activity in sensory regions [29], [30], [122]. The ventral bank of the superior temporal sulcus of the left hemisphere has been implicated in audio-visual speech integration [123] so it would be interesting to examine activation patterns in those with ASD in this region using functional imaging techniques.

It remains for future research to determine how reported abnormalities in brain development [22]–[33] relate to the auditory processing profile for ASD revealed in the present paper. However, hypotheses to explore include whether reduction in long-range connectivity leads to less top-down modulation of perceptual processes, which would affect the ability to filter out irrelevant information and the ability to decipher speech in noisy environments. Reduced long-distance connectivity might also be expected to make integration and synchronization between sensory regions difficult for those with ASD, consistent with decreased auditory-visual integration. Increased local connectivity might relate to the propensity of those with ASD to focus on details to a greater extent than for normally developing individuals, leading to categorical perception that is less specialized for sounds in the native environment and to the increased frequency of absolute pitch in ASD.

It is noteworthy that musical processing appears to be relatively preserved among those with ASD. Interestingly, the ability to detect small pitch changes was preserved in those with ASD, and this ability was positively related to pitch memory, metrical processing, and harmonic processing as well as native phoneme processing. Overall, our results suggest that music might be a powerful remediation tool. Indeed there are suggestions that individuals with autism are more attracted to music than to speech [109], [124], [125]. Perhaps the regular structure of music can provide a scaffold for the organization of sensory input [126]–[128]. Music also has added benefits for social development in that group music making can increase prosocial behavior, including co-operation and eye contact [129].

Finally, it is worth noting that there was considerable variability among those with ASD on some of the tasks, adding to the evidence that ASD manifests differently from individual to individual. For example, compared to controls, a high proportion of those with ASD (11%) had absolute pitch, but the other 89% appeared to process pitch similarly as controls. In the McGurk task, 9 out of 27 of those with ASD appeared to have normal auditory-visual speech integration, but the rest showed marked difference from the norm. It is important to understand these individual differences and how they develop. It is possible that more typical outcomes such as these are in part the result of particular experiences or training programs early in development, but it is impossible to determine this from the present data. That many of the impairments found in the ASD group were dependent upon abilities normally acquired during infancy suggests that there might be sensitive periods for the development of these abilities. Thus, future research is needed to determine whether there are sensitive periods during which these perceptual abnormalities can be best ameliorated through specific training.

## References

[1] Frith U (1989) Autism: Explaining the enigma. Oxford: Blackwell.

[2] Peppé S, McCann J, Gibbon F, O’Hare A, Rutherford M (2007) Receptive and expressive prosodic ability in children with high-functioning autism. Journal of Speech, Language, and Hearing Research 50: 1015–1028. 10.1044/1092-4388(2007/071) 17675602

[3] Dawson G, Meltzoff A, Osterling J, Rinaldi J, Brown E (1998) Children with autism fail to orient to naturally-occurring social stimuli. Journal of Autism and Developmental Disorders 28: 479–485. 9932234 10.1023/a:1026043926488

[4] Klin A, Lin DJ, Gorrindo P, Ramsay G, Jones W (2009) Literature review: Preference for physical compared to biological motion: Genome-wide association study. Autism Research 2: 178–179.

[5] Pierce K, Conant D, Hazin R, Stoner R, Desmond J (2010) Preference for geometric patterns early in life as a risk factor for autism. Archives of General Psychiatry 68: 101–109. 20819977 10.1001/archgenpsychiatry.2010.113 PMC4894313

[6] Rutherford MD (2005) A retrospective journal-based case study of the development of a child with autism and his twin. Neurocase 11: 1–9. 16036467 10.1080/13554790590925529

[7] Zwaigenbaum L, Bryson S, Lord C, Rogers S, Carter A, et al (2009) Clinical assessment and management of toddlers with suspected autism spectrum disorder: Insights from studies of high-risk infants. Pediatrics 123: 1383–1391. 19403506 10.1542/peds.2008-1606 PMC2833286

[8] Happé F, Frith U (2006) The weak coherence account: Detail-focused cognitive style in autism spectrum disorders. Journal of Autism and Developmental Disorders 35: 5–25. 10.1007/s10803-005-0039-0 16450045

[9] Baron-Cohen S, Hammer J (1997) Parents of children with asperger syndrome: What is the cognitive phenotype? Journal of Cognitive Neuroscience 9: 548–554. 23968217 10.1162/jocn.1997.9.4.548

[10] Plaisted K, Swettenham J, Rees L (1999) Children with autism show local precedence in a divided attention task and global precedence in a selective attention task. Journal of Child Psychology and Psychiatry 40: 733–742. 10433407

[11] Shah A, Frith U (1983) An islet of ability in autistic children. A research note. Journal of Child Psychology and Psychiatry and Applied Disciplines 24: 613–620. 10.1111/j.1469-7610.1983.tb00137.x 6630333

[12] Baranek GT, Boyd BA, Poe MD, David FJ, Watson LR (2007) Hyperresponsive sensory patterns in young children with autism, developmental delay, and development. American Journal of Mental Retardation 112: 233–245. 17559291 10.1352/0895-8017(2007)112[233:HSPIYC]2.0.CO;2

[13] Dahlgren SV, Gillberg C (1989) Arousal, attention, and the socioemotional impairments of individuals with autism. In: Dawson G, editor. Autism: Nature, diagnosis and treatment. New York: Guilford. 49–74.

[14] Gomes E, Rotta NT, Pedroso FS, Sleifer P, Danesi MC (2004) Auditory hypersensitivity in children and teenagers with autistic spectrum disorder. Arq Neuropsiquiatr 62: 797–801. 15476072 10.1590/s0004-282x2004000500011

[15] Kern JK, Trivedi MH, Garver CR, Grannemann BD, Andrews AA, et al (2006) The pattern of sensory processing abnormalities in autism. Autism 10: 480–490. 16940314 10.1177/1362361306066564

[16] Rimland B, Edelson SM (1995) Brief report: A pilot study of auditory integration training in autism. Journal of Autism and Developmental Disabilities 25: 61–70. 10.1007/BF02178168 7608035

[17] Rosenhall U, Nordin V, Sandstrom M, Ahlsen G, Gillberg C (1999) Autism and hearing loss. Journal of Autism and Developmental Disorders 29: 349–357. 10.1023/a:1023022709710 10587881

[18] Baranek GT (1999) Autism during infancy: A retrospective video analysis of sensory-motor and social behaviors at 9–12 months of age. Journal of Autism and Developmental Disorders 29: 213–224. 10425584 10.1023/a:1023080005650

[19] Dawson G, Toth K, Abbott R, Osterling J, Munson J, et al (2004) Early social attention impairments in autism: Social orienting, joint attention, and attention to distress. Developmental Psychology 40: 271–83. 14979766 10.1037/0012-1649.40.2.271

[20] Osterling J, Dawson G (1994) Early recognition of children with autism: A study of first birthday home video tapes. Journal of Autism and Developmental Disorders 24: 247–257. 8050980 10.1007/BF02172225

[21] Werner E, Dawson G, Osterling J, Dinno N (2000) Brief report: Recognition of autism spectrum disorder before one year of age: A retrospective study based on home videotapes. Journal of Autism and Developmental Disorders 30: 157–162. 10832780 10.1023/a:1005463707029

[22] Courchesne E, Karns CM, Davis HR, Ziccardi R, Carper RA, et al (2001) Unusual brain growth patterns in early life in patients with autistic disorder: An MRI study. Neurology 57: 245–254. 11468308 10.1212/wnl.57.2.245

[23] Courchesne E, Carper R, Akshoomoff N (2003) Evidence of brain overgrowth in the first year of life in autism. The Journal of the American Medical Association 290: 337–344. 12865374 10.1001/jama.290.3.337

[24] Courchesne E (2004) Brain development in autism: Early overgrowth followed by premature arrest of growth. Mental Retardation and Developmental Disability Research Review 10: 106–111. 10.1002/mrdd.20020 15362165

[25] Casanova MF (2004) Intracortical circuitry: One of Psychiatry’s missing assumptions. European Archives of Psychiatry and Clinical Neuroscience 254: 148–151. 15205967 10.1007/s00406-004-0457-6

[26] Casanova MF, Buzhoeveden DP, Switala AE, Roy E (2002) Minicolumnar pathology in autism. American Academy of Neurology 58: 428–432. 10.1212/wnl.58.3.428 11839843

[27] Courchesne E, Pierce K (2005) Why the frontal cortex in autism might be talking only to itself: Local over-connectivity but long-distance disconnection. Current Opinion in Neurobiology 15: 225–230. 15831407 10.1016/j.conb.2005.03.001

[28] Barnea-Goraly N, Kwon H, Menon V, Eliez S, Lotspeich L, et al (2004) White matter structure in autism: Preliminary evidence from diffusion tensor imaging. Biological Psychiatry 55: 323–326. 14744477 10.1016/j.biopsych.2003.10.022

[29] Bird G, Catmur C, Silani G, Frith C, Frith U (2006) Attention does not modulate neural responses to social stimuli in autism spectrum disorders. Neuroimage 31: 1614–1624. 16616862 10.1016/j.neuroimage.2006.02.037

[30] Castelli F, Frith C, Happé F, Frith U (2002) Autism, asperger syndrome and brain mechanisms for the attribution of mental states to animated shapes. Brain 125: 1839–1849. 12135974 10.1093/brain/awf189

[31] Just MA, Cherkassky VL, Keller TA, Kana RK, Minshew NJ (2007) Functional and anatomical cortical underconnectivity in autism: Evidence from an fMRI study of an executive function task and corpus callosum morphometry. Cerebral Cortex 17: 951–961. 16772313 10.1093/cercor/bhl006 PMC4500121

[32] Just MA, Cherkassky VL, Keller TA, Minshew NJ (2004) Cortical activation and synchronization during sentence comprehension in high-functioning autism: Evidence of underconnectivity. Brain 127: 1811–1821. 15215213 10.1093/brain/awh199

[33] Skukla DK, Keehn B, Müller R (2010) Tract-specific analyses of diffusion tensor imaging show widespread white matter compromise in autism spectrum disorder. Journal of Child Psychology and Psychiatry 52: 286–295. 21073464 10.1111/j.1469-7610.2010.02342.x PMC4547854

[34] Griffiths TD (2007) Sorting out Sound. Neuron 56: 580–581. 18031677 10.1016/j.neuron.2007.11.004

[35] Boddaert N, Chabane N, Belin P, Bourgeois M, Royer V, et al (2004) Perception of complex sounds in autism: Abnormal auditory cortical processing in children. American Journal of Psychiatry 161: 2117–2120. 15514415 10.1176/appi.ajp.161.11.2117

[36] Herbert MR, Harris GJ, Adrien KT, Ziegler DA, Makris N, et al (2002) Abnormal asymmetry in language association cortex in autism. Annals of Neurology 52: 588–596. 12402256 10.1002/ana.10349

[37] Ashburner J, Ziviani J, Rodger S (2008) Sensory processing and classroom emotional behavioural, and education outcomes in children with autism spectrum disorder. American Journal of Occupational Therapy 62: 564–573. 18826017 10.5014/ajot.62.5.564

[38] Baker AE, Lane A, Angley MT, Young RL (2008) The relationship between sensory processing patterns and behavioural responsiveness in autistic disorder: A pilot study. Journal of Autism and Developmental Disorders 38: 867–875. 17899349 10.1007/s10803-007-0459-0

[39] Lane A, Young RL, Baker AEZ, Angley M (2010) Sensory processing subtypes in autism: Association with adaptive behavior. Journal of Autism and Developmental Disorders 40: 112–122. 19644746 10.1007/s10803-009-0840-2

[40] Rogers SJ, Hepburn S, Wehner E (2003) Parent reports of sensory symptoms in toddlers with autism and those with other developmental disorders. Journal of Autism and Developmental Disorders 33: 631–42. 14714932 10.1023/b:jadd.0000006000.38991.a7

[41] Schoen SA, Miller LJ, Brett-Green BA, Nielsen DM (2009) Physiological and behavioral differences in sensory processing: A comparison of children with autism spectrum disorder and sensory modulation disorder. Frontiers in Integrative Neuroscience 3: 1–11. 19915733 10.3389/neuro.07.029.2009 PMC2776488

[42] McIntosh DN, Miller LJ, Shyu V, Dunn W (1999) Overview of the Short Sensory Profile (SSP). In: Dunn W, editor. The sensory profile: Examiner’s manual. San Antonio, TX: The Psychological Corporation. 59–73.

[43] Alcántara JI, Weisblatt EJL, Moore BCJ, Bolton PF (2004) Speech-in-noise perception in high-functioning individuals with autism or asperger’s syndrome. Journal of Child Psychology and Psychiatry 45: 1107–1114. 15257667 10.1111/j.1469-7610.2004.t01-1-00303.x

[44] Groen WB, van Orsouw L, ter Huurne N, Swinkels S, van der Gaag RJ, et al (2009) Intact spectrum but abnormal temporal processing of auditory stimuli in autism. Journal of Autism and Developmental Disorders 39: 742–750. 19148738 10.1007/s10803-008-0682-3

[45] Teder-Sälejärvi WA, Pierce KL, Courchesne E, Hillyard SA (2005) Auditory spatial localization and attention deficits in autistic adults. Cognitive Brain Research 23: 221–234. 15820630 10.1016/j.cogbrainres.2004.10.021

[46] Lepistö T, Kuitunen A, Sussman E, Saalasti S, Jansson-Verkasalo E, et al (2009) Auditory stream segregation in children with asperger syndrome. Biological Psychology 82: 301–307. 19751798 10.1016/j.biopsycho.2009.09.004 PMC2771139

[47] Russo N, Nicol T, Trommer B, Zecker S, Kraus N (2009) Brainstem transcription of speech is disrupted in children with autism spectrum disorders. Developmental Science 12: 557–567. 19635083 10.1111/j.1467-7687.2008.00790.x PMC2718770

[48] Russo NM, Zecker S, Trommer B, Chen J, Kraus N (2009) Effects of background noise on cortical encoding of speech in autism spectrum disorders. Journal of Autism and Developmental Disorders 39: 1185–1196. 19353261 10.1007/s10803-009-0737-0 PMC2810203

[49] Best CT, McRoberts GW, Goodell E (2001) Discrimination of non-native consonant contrasts varying in perception assimilation to the listener’s native phonological system. Journal of Acoustic Society of America 109: 775–794. 10.1121/1.1332378 PMC2777975 11248981

[50] Werker JF, Tees RC (2005) Speech perception as a window for understanding plasticity and commitment in language systems of the brain. Developmental Psychobiology 46: 233–251. 15772961 10.1002/dev.20060

[51] Werker JF, Lalonde CE (1988) Cross-language speech perception: Initial capabilities and developmental change. Developmental Psychology 24: 672–683.

[52] Gopnik A, Meltzoff AN, Kuhl PK (1999) The scientist in the crib: Minds, brains and how children learn. New York: Harper Collins.

[53] Kuhl PK, Tsao FM, Liu HM (2003) Foreign-language experience in infancy: Effects of short-term exposure and social interaction on phonetic learning. Proceedings of the National Academy of Sciences 100: 9096–9101. 10.1073/pnas.1532872100 PMC166444 12861072

[54] Dawson G, Webb SJ, McPartland J (2005) Understanding the nature of face processing impairment in autism: Insights from behavioral and electrophysiological studies. Developmental Neuropsychology 27: 403–424. 15843104 10.1207/s15326942dn2703_6

[55] Osterling JA, Dawson G, Munson JA (2002) Early recognition of 1-year-old infants with autism spectrum disorder versus mental retardation. Developmental Psychopathology 14: 239–251. 10.1017/s0954579402002031 12030690

[56] Schultz RT, Gauthier I, Klin A, Fulbright RK, Anderson AW, et al (2000) Abnormal ventral temporal cortical activity during face discrimination among individuals with autism and Asperger syndrome. Archives of General Psychiatry 57: 331–340. 10768694 10.1001/archpsyc.57.4.331

[57] Constantino JN, Yang D, Gray TL, Gross MM, Abbacchi AM, et al (2007) Clarifying the association between language and social development in autism: A study of non-native phoneme recognition. Journal of Autism and Developmental Disorders 37: 1256–1263. 17080273 10.1007/s10803-006-0269-9

[58] McGurk H, MacDonald J (1976) Hearing lips and seeing voices. Nature 264: 746–748. 1012311 10.1038/264746a0

[59] De Gelder B, Vroomen J, van der Heide L (1991) Face recognition and lip-reading in autism. European Journal of Cognitive Psychology 3: 69–86.

[60] Iarocci G, Rombough A, Yager J, Weeks DJ, Chua R (2010) Visual inﬂuences on speech perception in children with autism. Autism 14: 305–320. 20591957 10.1177/1362361309353615

[61] Williams JHG, Massaro DW, Peel NJ, Bosseler A, Suddendorf T (2004) Visual auditory integration during speech imitation in autism. Research in Developmental Disabilities 25: 559–575. 15541632 10.1016/j.ridd.2004.01.008

[62] Smith EG, Bennetto L (2007) Audiovisual speech integration and lipreading in autism. Journal of Child Psychology and Psychiatry 48: 813–821. 17683453 10.1111/j.1469-7610.2007.01766.x

[63] Plantinga J, Trainor LJ (2008) Infants’ memory for isolated tones and the effects of interference. Music Perception 26: 121–128.

[64] Trainor LJ, Trehub SE (1992) A comparison of infants’ and adults’ sensitivity to Western musical structure. Journal of Experimental Psychology: Human Perception and Performance 18: 394–402. 1593226 10.1037//0096-1523.18.2.394

[65] Trehub SE (2001) Musical predispositions in infancy. Annals of the New York Academy of Sciences 930: 1–16. 10.1111/j.1749-6632.2001.tb05721.x 11458822

[66] Trehub SE, Bull D, Thorpe LA (1984) Infants’ perception of melodies: The role of melodic contour. Child Development 55: 821–830. 6734320 10.1111/j.1467-8624.1984.tb03819.x

[67] Bachem A (1955) Absolute pitch. Journal of the Acoustical Society of America 27: 1180–1185.

[68] Brown WA, Cammuso K, Sachs H, Winklosky B, Mullane J, et al (2003) Autism-related language, personality, and cognition in people with absolute pitch: Results of a preliminary study. Journal of Autism and Developmental Disorders 33: 163–167. 12757355 10.1023/a:1022987309913

[69] Brenton JN, Devries SP, Barton C, Minnich H, Sokol D (2008) Absolute pitch in a four-year-old boy with autism. Pediatric Neurology 39: 137–138. 18639762 10.1016/j.pediatrneurol.2008.05.004

[70] Heaton P, Pring L, Hermelin B (1999) A pseudo-savant: A case of exceptional musical splinter skills. Neurocase 5: 503–509.

[71] Mottron L, Peretz I, Belleville S, Rouleau N (1999) Absolute pitch in autism: A case-study. Neurocase 5: 485–501.

[72] Young RL, Nettelbeck T (1995) The abilities of a musical savant and his family. Journal of Autism and Developmental Disorders 25: 231–248. 7559290 10.1007/BF02179286

[73] Hannon EE, Trainor LJ (2007) Music acquisition: Effects of enculturation and formal training on development. Trends in Cognitive Sciences 11: 466–472. 17981074 10.1016/j.tics.2007.08.008

[74] Trainor LJ, Corrigall KA (2010) Music acquisition and effects of musical experience. In: Riess-Jones M, Fay RR, editors. Springer Handbook of Auditory Research: Music Perception. Heidelberg: Springer. 89–128.

[75] Hannon EE, Trehub SE (2005a) Metrical categories in infancy and adulthood. Psychological Science 16: 48–55. 15660851 10.1111/j.0956-7976.2005.00779.x

[76] Hannon EE, Trehub SE (2005b) Tuning in to musical rhythms: Infants learn more readily than adults. Proceedings of the National Academy of Sciences 102: 12639–12643. 10.1073/pnas.0504254102 PMC1194930 16105946

[77] Trehub SE, Hannon EE (2009) Conventional rhythms enhance infants’ and adults’ perception of music. Cortex 45: 110–118. 19058799 10.1016/j.cortex.2008.05.012

[78] Trainor LJ, Trehub SE (1994) Key membership and implied harmony in Western tonal music: Developmental perspectives. Perception and Psychophysics 56: 125–132. 7971113 10.3758/bf03213891

[79] Clayton M (2000) Time in Indian music. New York: Oxford University Press.

[80] Gerry DW, Faux AL, Trainor LJ (2010) Effects of Kindermusik training on infants’ rhythmic enculturation. Developmental Science 13: 545–551. 20443974 10.1111/j.1467-7687.2009.00912.x

[81] Corrigall KA, Trainor LJ (2010) Musical enculturation in preschool children: Acquisition of key and harmonic knowledge. Music Perception 28: 195–200.

[82] Schellenberg EG, Bigand E, Poulin-Charronnat B, Garnier C, Stevens C (2005) Children’s implicit knowledge of harmony in Western music. Developmental Science 8: 551–566. 16246247 10.1111/j.1467-7687.2005.00447.x

[83] Bigand E, Madurell F, Tillmann B, Pineau M (1999) Effect of global structure and temporal organization on chord processing. Journal of Experimental Psychology: Perception and Performance 25: 184–197.

[84] Poulin-Charronnat B, Bigand E, Madurell F, Peereman R (2004) Musical structure modulates semantic priming in vocal music. Cognition 94: B67–B78. 10.1016/j.cognition.2004.05.003 15617668

[85] Tillmann B, Bigand E, Escoffier N, Lalitte P (2006) The inﬂuence of musical relatedness on timbre discrimination. European Journal of Cognitive Psychology 18: 343–358.

[86] Tillmann B, Peretz I, Bigand E, Gosselin N (2007) Harmonic priming in an amusic patient: The power of implicit tasks. Cognitive Neuropsychology 24: 1–20. 10.1080/02643290701609527 18416511

[87] Heaton P, Williams K, Cummins O, Happe F (2007) Beyond perception: Musical representation and on-line processing in autism. Journal of Autism and Developmental Disorders 37: 1355–1360. 17146705 10.1007/s10803-006-0283-y

[88] Lord C, Rutter M, Goode S, Heemsbergen J, Jordan H, et al (1989) Autism diagnostic observation schedule: A standardized observation of communicative and social behavior. Journal of Autism and Developmental Disorders 19: 185–212. 2745388 10.1007/BF02211841

[89] Lord C, Rutter M, Le Couteur A (1994) Autism Diagnostic Interview-Revised: A revised version of a diagnostic interview for caregivers of individuals with possible pervasive developmental disorders. Journal of Autism and Developmental Disorders 24: 659–685. 7814313 10.1007/BF02172145

[90] Pfordresher PQ, Brown S (2009) Poor-pitch singing in the absence of “tone deafness.”. Music Perception 25: 95–115.

[91] Ross DA, Olson IR, Marks LE, Gore JC (2004) A nonmusical paradigm for identifying absolute pitch possessors. Journal of the Acoustical Society of America 116: 1793–1799. 15478446 10.1121/1.1758973

[92] Wechsler D (1997) Digit Span Subtest of the Wechsler Memory Scale-III. San Antonio, TX: The Psychological Corporation.

[93] Dunn LM, Dunn LM (1997) Peabody Picture Vocabulary Test (3rd ed) Circle Pines, MN: American Guidance Services.

[94] Willeford JA (1977) Assessing central auditory behavior in children: A test battery approach. In: Keith R, editor. Central auditory dysfunction. New York: Grune & Stratton. 43–72.

[95] Roid G, Miller L (2005) Leiter International Performance Scale. GL Assessment: London.

[96] Tucker-Davis Technologies (2007) Programmable attenuator. Alachua, FL: Tucker-Davis Technologies.

[97] Han LA, Poulsen T (1998) Equivalent threshold sound pressure levels (ETSPL) for sennheiser HDA 200 earphone and etymotic research ER-2 insert earphone in the frequency range 125 Hz to 16 kHz. Scandinavian Audiology 27: 105–112. 9638829 10.1080/010503998420342

[98] Neurobehavioral Systems (2007) Presentation 11.0. Albany, CA: Neurobehavioral Systems, Incorporated.

[99] Watson AB, Pelli DG (1983) QUEST: A Bayesian adaptive psychometric method. Perception & Psychophysics 33: 113–120. 6844102 10.3758/bf03202828

[100] Ratcliff R (1993) Methods for dealing with reaction time outliers. Psychological Bulletin 114: 510–532. 8272468 10.1037/0033-2909.114.3.510

[101] Patel AD, Iversen JR, Bregman MR, Schulz I (2009) Experimental evidence for synchronization to a musical beat in a nonhuman animal. Current Biology 19: 827–830. 19409790 10.1016/j.cub.2009.03.038

[102] Schachner A, Brady TF, Pepperberg I, Hauser M (2009) Spontaneous motor entrainment to music in multiple vocal-mimicking species. Current Biology 19: 831–836. 19409786 10.1016/j.cub.2009.03.061

[103] Kirschner S, Tomasello M (2009) Joint drumming: Social context facilitates synchronization in preschool children. Journal of Experimental Child Psychology 102: 299–314. 18789454 10.1016/j.jecp.2008.07.005

[104] Kirschner S, Tomesello M (2010) Joint music making promotes prosocial behaviour in 4-year-old children. Evolution and Human Behaviour 31: 354–364.

[105] Allen R, Hill E, Heaton P (2009) ‘Hath charms to soothe…’: An exploratory study of how high-functioning adults with ASD experience music. Autism 13: 21–41. 19176575 10.1177/1362361307098511

[106] Costa-Giomi E (2003) Young children’s harmonic perception. Annals of the New York Academy of Sciences 999: 477–484. 14681171 10.1196/annals.1284.058

[107] Heaton P (2009) Assessing musical skills in autistic children who are not savants. Philosophical Transactions of the Royal Society B: Biological Sciences 364: 1443–1447. 10.1098/rstb.2008.0327 PMC2677585 19528029

[108] Heaton P, Hudry K, Ludlow A, Hill EL (2008) Superior discrimination of speech pitch and its relationship to verbal ability in autism spectrum disorders. Cognitive Neuropsychology 25: 771–782. 18720290 10.1080/02643290802336277

[109] Jarvinen-Pasley A, Heaton P (2007) Evidence for reduced domain-specificity in auditory processing in autism. Developmental Science 10: 786–793. 17973796 10.1111/j.1467-7687.2007.00637.x

[110] Rimland B, Hill AL (1984) Idiot savants. In: Wortis J, editor. Mental Retardation and Developmental Disabilities. New York: Plenum Press. 55–169.

[111] Baharloo S, Johnston PA (1998) Service SK, Gitschier J, Freimer NB (1998) Absolute pitch: An approach for identification of genetic and nongenetic components. Journal of Human Genetics 62: 224–231. 10.1086/301704 PMC1376881 9463312

[112] Loui P, Li H, Hohmann A, Schlaug G (2011) Enhanced cortical connectivity in absolute pitch musicians: A model for local hyperconnectivity. Journal of Cognitive Neuroscience 23: 1015–1026. 20515408 10.1162/jocn.2010.21500 PMC3012137

[113] Folland N, Butler BE, Smith NA, Trainor LJ (2012) Processing simultaneous auditory objects in infancy: Music and mistuned harmonics. Journal of the Acoustical Society of America 131: 993–997. 22280722 10.1121/1.3651254

[114] Demany L (1982) Auditory stream segregation in infancy. Infant Behavior & Development 48: 261–276.

[115] Fassbender C (1993) Auditory grouping and segregation processes in infancy. Norderstedt: Kaste Verlag.

[116] McAdams S, Bertoncini J (1997) Organization and discrimination of repeating sound sequences by newborn infants. Journal of the Acoustical Society of America 102: 2945–2953. 9373981 10.1121/1.420349

[117] Smith NA, Trainor LJ (2011) Auditory stream segregation improves infants’ selective attention to target tones amid distracters. Infancy 16: 1–14. 10.1111/j.1532-7078.2011.00067.x PMC3203020 22039336

[118] Winkler I, Kushnerenko E, Horvath J, Ceponiene R, Fellman V, et al (2003) Newborn infants can organize the auditory world. Proceedings of the National Academy of Sciences of the United States of America 100: 11812–11815. 14500903 10.1073/pnas.2031891100 PMC208846

[119] Sussman E, Wong R, Horváth J, Winkler I, Wang W (2007) The development of perceptual organization of sound by frequency separation in 5–11 year-old children. Hearing Research 225: 117–127. 17300890 10.1016/j.heares.2006.12.013

[120] Kawabe T, Shirai N, Wada Y, Miura K, Kanazawa S, et al (2010) The audiovisual tau effect in infancy. PLoS ONE 5: 1–7. 10.1371/journal.pone.0009503 PMC2831064 20209137

[121] Rosenblum LD, Schmuckler MA, Johnson JA (1997) The McGurk effect in infants. Perceptual Psychophysics 59: 347–357. 10.3758/bf03211902 9136265

[122] Iarocci G, McDonald J (2006) Sensory integration and the perceptual experience of persons with autism. Journal of Autism and Developmental Disorders 36: 77–90. 16395537 10.1007/s10803-005-0044-3

[123] Calvert GA, Campbell R, Brammer MJ (2000) Evidence from functional magnetic resonance imaging of crossmodal binding in the human heteromodal cortex. Current Biology 10: 649–657. 10837246 10.1016/s0960-9822(00)00513-3

[124] Finnigan E, Starr E (2010) Increasing social responsiveness in a child with autism: A comparison of music and non-music interventions. Autism 14: 321–348. 20591958 10.1177/1362361309357747

[125] Simpson K, Keen D (2010) Teaching young children with autism graphic symbols embedded within an interactive song. Journal of Developmental and Physical Disabilities 22: 165–177.

[126] Allen R, Heaton P (2010) Autism, music, and the therapeutic potential of music in alexithymia. Music Perception 27: 251–261.

[127] Wan CY, Demaine K, Zipse L, Norton A, Schlaug G (2010) From music making to speaking: Engaging the mirror neuron system in autism. Brain Research Bulletin 82: 161–168. 20433906 10.1016/j.brainresbull.2010.04.010 PMC2996136

[128] Wan CY, Schlaug G (2010) Neural pathways for language in autism: The potential for music-based treatments. Future Neurology 5: 797–805. 21197137 10.2217/fnl.10.55 PMC3011184

[129] Overy K, Molnar-Szakacs I (2009) Being together in time: Musical experience and the mirror neuron system. Music Perception 26: 489–504.

[130] Bigand E, Poulin B, Tillmann B, Madurell F, D’Adamo DA (2003) Sensory versus cognitive components in harmonic priming. Journal of Experimental Psychology 29: 159–171. 12669755 10.1037//0096-1523.29.1.159

